# Supplementary material for: The AML cellular state space unveils NPM1 immune evasion subtypes with distinct clinical outcomes
Source: Nat Commun. 2025 Nov 25;16:10592. doi: 10.1038/s41467-025-66546-6 (PMC12658069; doi:10.1038/s41467-025-66546-6)
Supplement: Supplementary file 1 — Supplementary Information [file 41467_2025_66546_MOESM1_ESM.pdf]

SUPPLEMENTARY INFORMATION

The AML cellular state space unveils *NPM1* immune evasion subtypes with distinct clinical outcomes

Henrik Lilljebjörn[1], Pablo Peña-Martínez[1], Hanna Thorsson[1], Rasmus Henningsson[1], Marianne Rissler[1], Niklas Landberg[1], Noelia Puente-Moncada[1], Sofia von Palffy[1], Vendela Rissler[1], Petr Stanek[1], Jonathan Desponds[2,3], Xiangfu Zhong[4], Gunnar Juliusson[5], Vladimir Lazarevic[5], Sören Lehmann[4], Magnus Fontes[2], Helena Ågerstam[1,6], Carl Sandén[1], Christina Orsmark-Pietras[1,6], Thoas Fioretos[1,6,7]

[1]Division of Clinical Genetics, Department of Laboratory Medicine, Lund University, Lund, Sweden.
[2]Institut Roche, Boulogne-Billancourt, France.
[3]Symphogen, Ballerup, Denmark.
[4]Department of Medicine, Center for Hematology and Regenerative Medicine, Karolinska Institute, Huddinge, Sweden.
[5]Department of Hematology, Oncology and Radiation Physics, Skåne University Hospital, Lund, Sweden.
[6]Department of Clinical Genetics, Pathology, and Molecular Diagnostics, Skåne University Hospital, Region Skåne, Lund, Sweden.
[7]Clinical Genomics Lund, Science for Life Laboratory, Lund University, Lund, Sweden

SUPPLEMENTARY INFORMATION

Supplementary figures
Supplementary references

Supplementary Figures

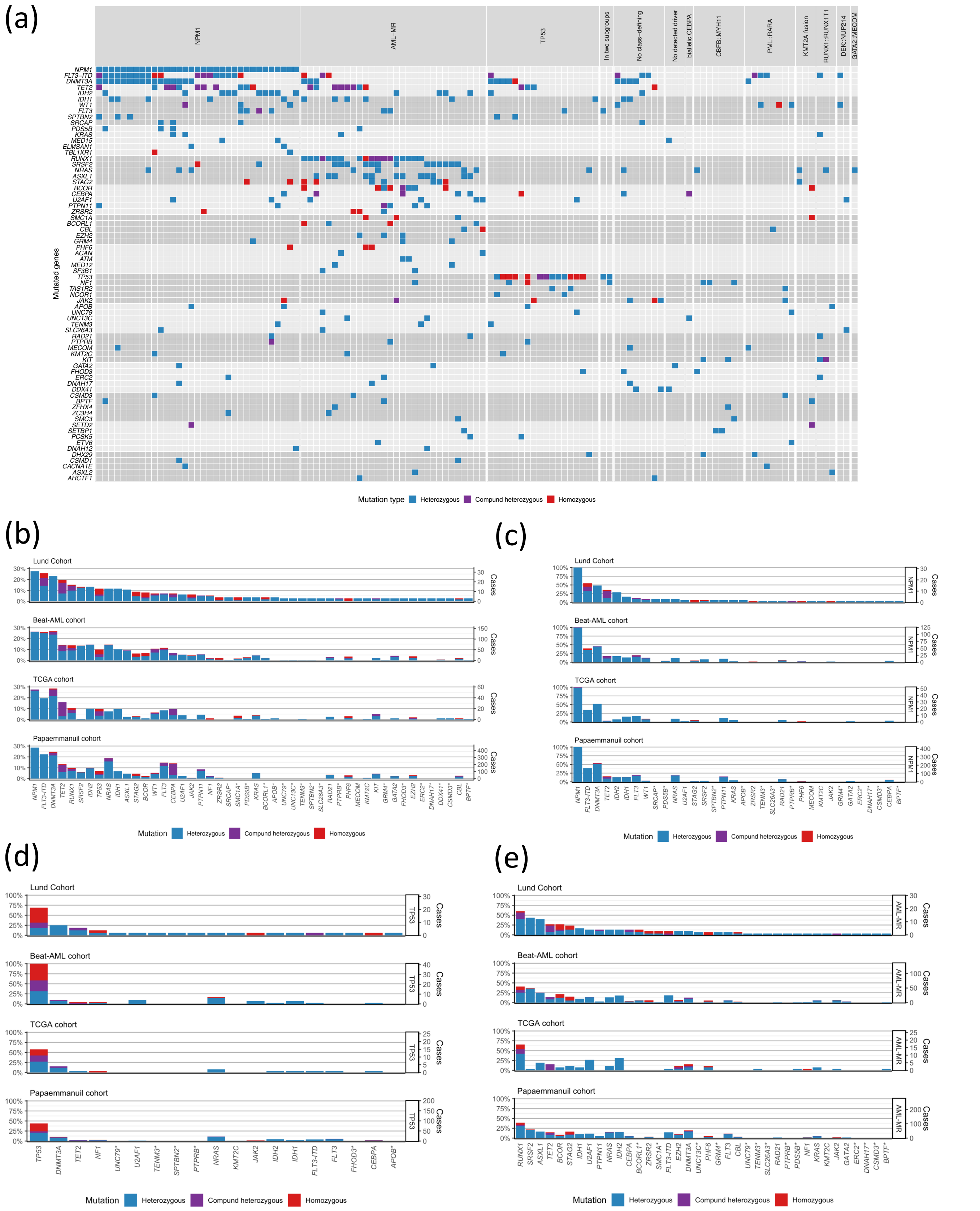

Supplementary Fig. 1 | Somatic mutations in AML. (a) Small nucleotide variants and insertion/deletions in the 70 most commonly mutated genes for the 120 AMLs included in the analyzed cohort. **(b)** Overall mutation frequencies compared with the AML cohorts from TCGA (n=200 AMLs),[1] Beat-AML (n=509 AMLs),[2] and Papaemmanuil et al (n=1540 AMLs). [3] **(c)** Mutation frequencies among *NPM1*-mutated cases compared with the AML cohorts from TCGA, Beat-AML, and Papaemmanuil et al. **(d)** Mutation frequencies among *TP53*-mutated cases compared with the AML cohorts from TCGA, Beat-AML, and Papaemmanuil et al. **(e)** Mutation frequencies among AML-MR cases compared with the AML cohorts from TCGA, Beat-AML, and Papaemmanuil et al. Source data are provided as a Source Data file.

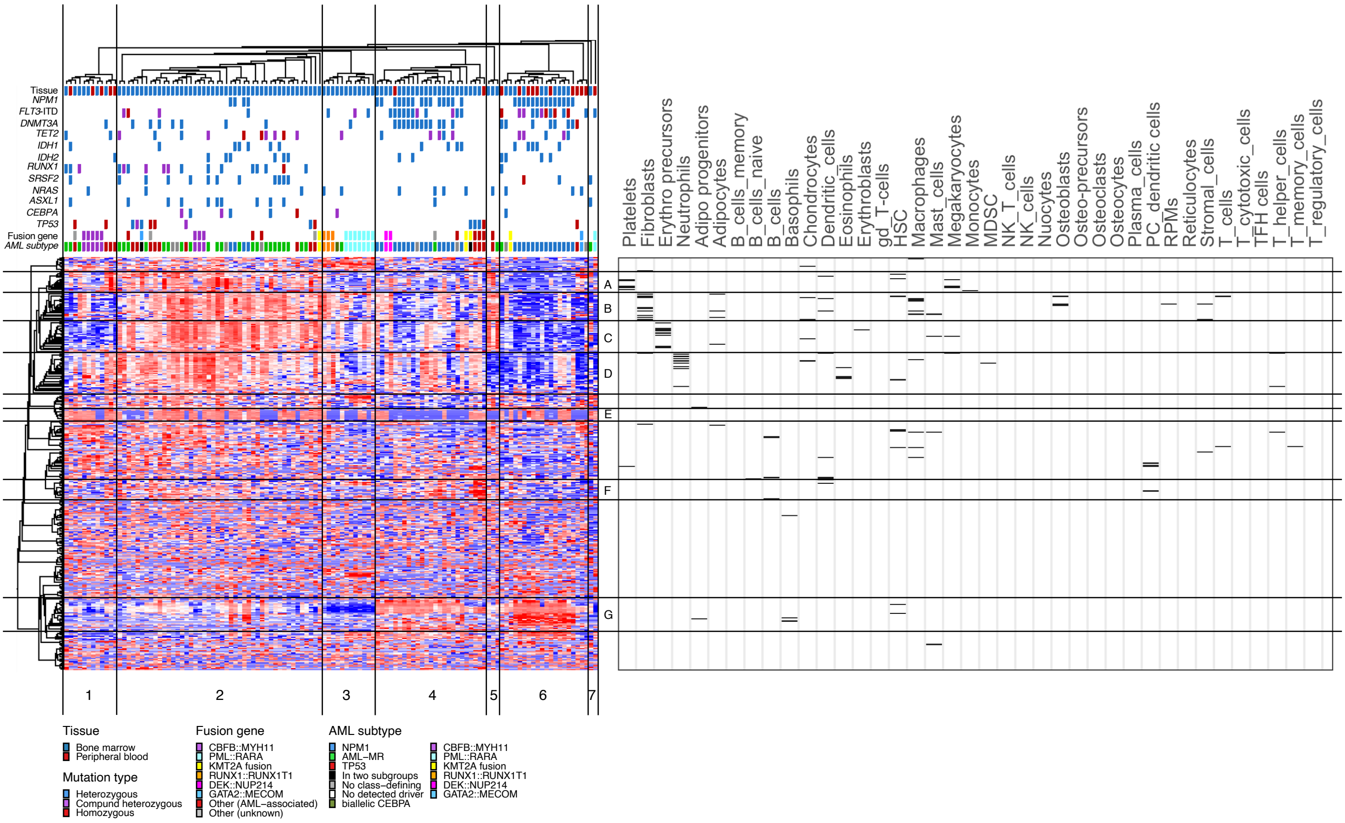

Supplementary Fig. 2 | Cell type markers are enriched among the genes with most variable expression in AML. Left side: Hierarchical clustering of the 120 AML samples based on gene expression of the 371 most variable genes (s/smax = 0.45). Distinct gene clusters are indicated by letters on the right side of the heatmap based on the gene dendogram. Right side: Genes within the heatmap that match cell type marker genes in PanglaoDB[4] for 39 distinct cell types present in blood, bone, connective tissue, or the immune system are indicated by black lines. In gene cluster A, 5/19 genes (26%) match marker genes for platelets. In cluster B, 9/25 genes (36%) match marker genes for fibroblasts. In gene cluster C, 8/26 genes (30%) match marker genes for erythroid-like and erythroid precursor cells. In gene cluster D, 8/33 genes (24%) match marker genes for neutrophils.

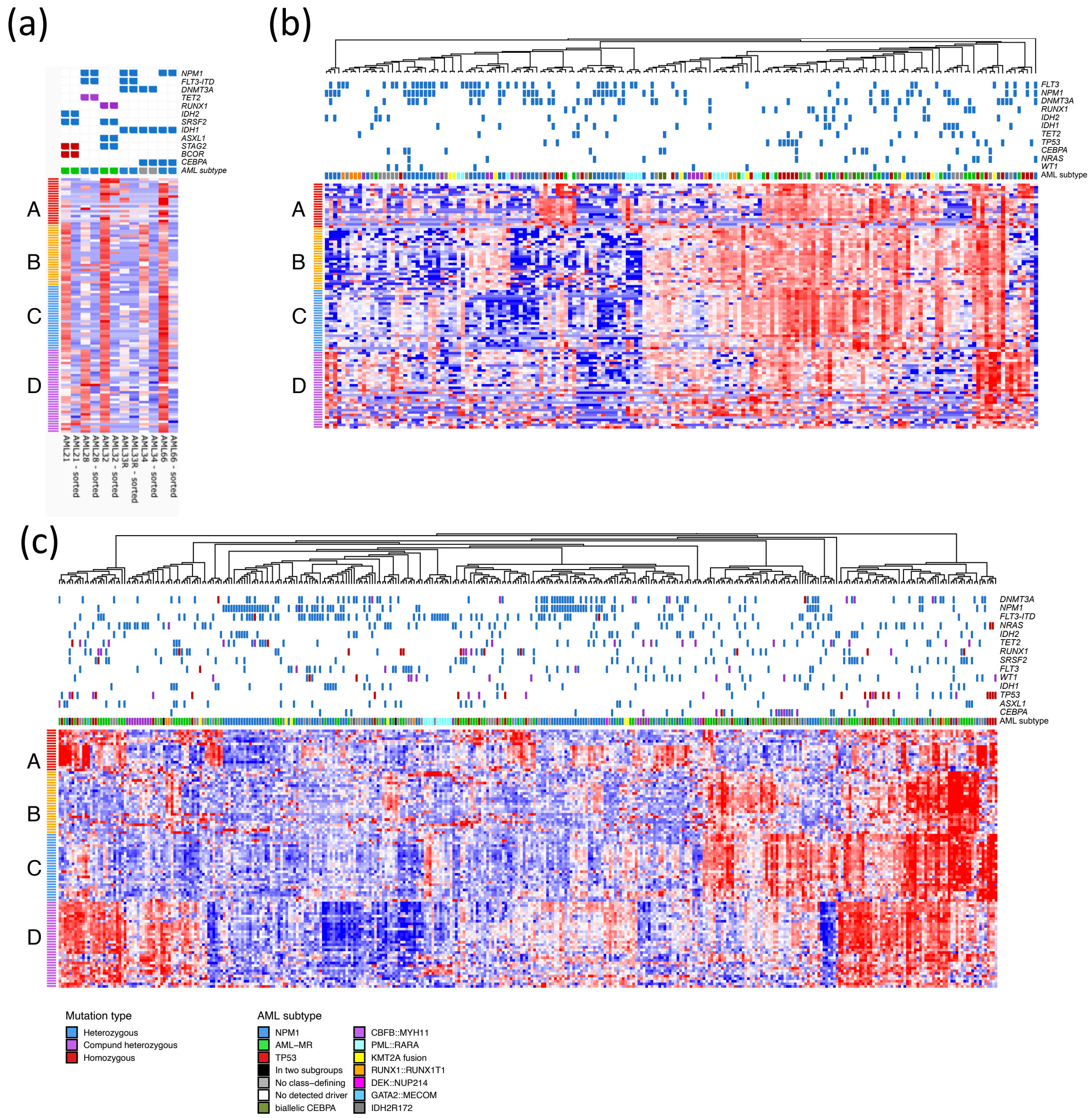

Supplementary Fig. 3 | Cell type markers in sorted samples and external datasets. (a) Heatmap illustrating the expression level of genes in the four gene clusters enriched for cell type markers (A,B,C, and D). Six bulk samples (AML21, AML28, AML32, AML33R, AML34, and AML66) and six samples sorted to contain only myeloid (CD33+/CD19-/CD3-) mononuclear cells (indicated with "- sorted") are included. **(b)** Heatmap illustrating the expression level of genes in the four gene clusters enriched for cell type markers (A,B,C, and D) in the TCGA[1] cohort (n=173 samples) **(c)** Heatmap illustrating the expression level of genes in the four gene clusters enriched for cell type markers (A,B,C, and D) in the the Beat-AML[2] cohort (n=360 samples).

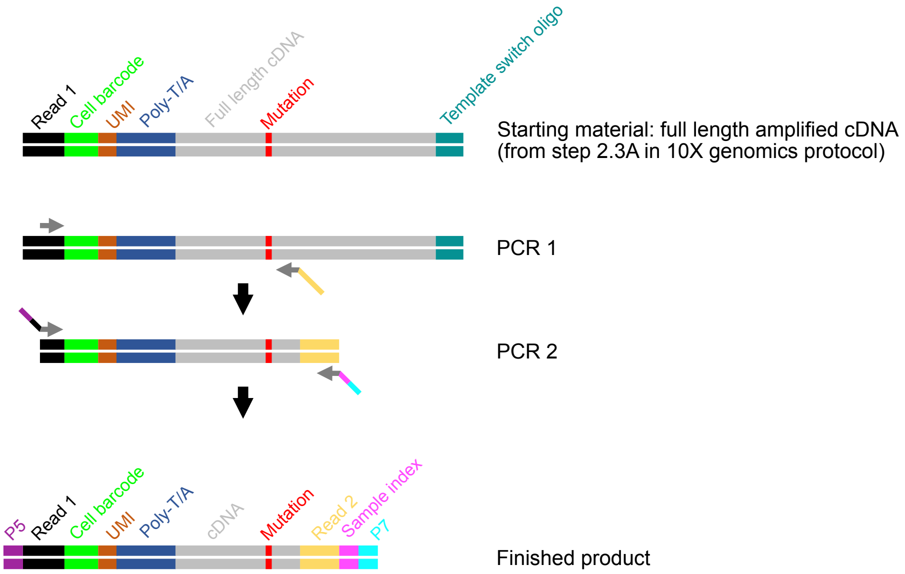

Supplementary Fig. 4 | Overview of mutation calling PCR strategy. Single cell mutation calling on scRNA-seq material was performed using a two-step PCR-amplification protocol. Full length amplified cDNA from an intermediate step in the 10X genomics chromium single cell 3´ v3 library preparation (step 2.3A) was used as PCR-template. In the first PCR, the right-hand primer was placed less than 100 bp from the targeted mutation. A 34-bp overhang (yellow) was added to the right-hand primer sequence. The left-hand primer for the first PCR was placed within the illumina Read1 sequence (black), thereby retaining the cell barcode and unique molecule identifier (UMI) information within the amplified material. In the second PCR, general primers binding to the left-hand Read1 sequence (black) and the right-hand Read2 sequence (yellow) were utilized, with overhang sequences adding sample index information (pink) and the P5 and P7 adapters (purple and turquoise) required for illumina sequencing. At sequencing, read 1 provides cell barcode and UMI information while read 2 provides mutation status for the targeted mutation.

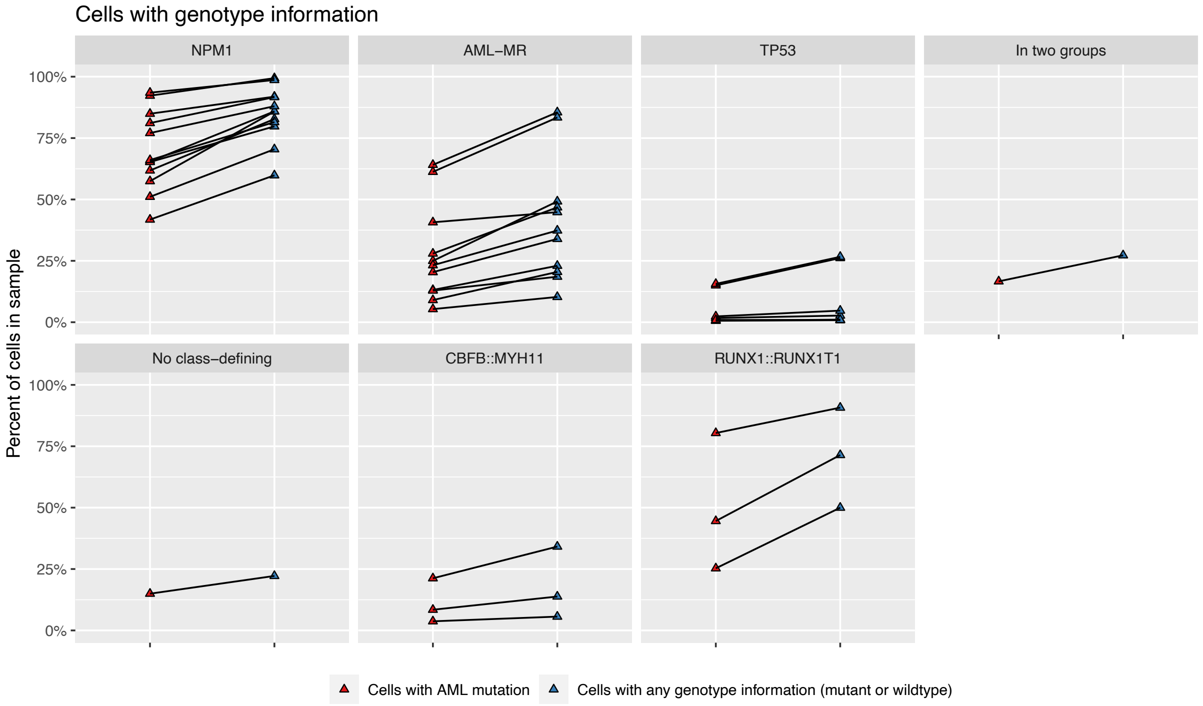

Supplementary Fig. 5 | Proportion of cells with genotype information. The proportion of cells with a detected AML mutation (red) and the proportion of cells with any genotype information (either mutant or wildtype) for the targeted positions (blue) illustrated for each sample. The samples are grouped by AML subtype. Source data are provided as a Source Data file.

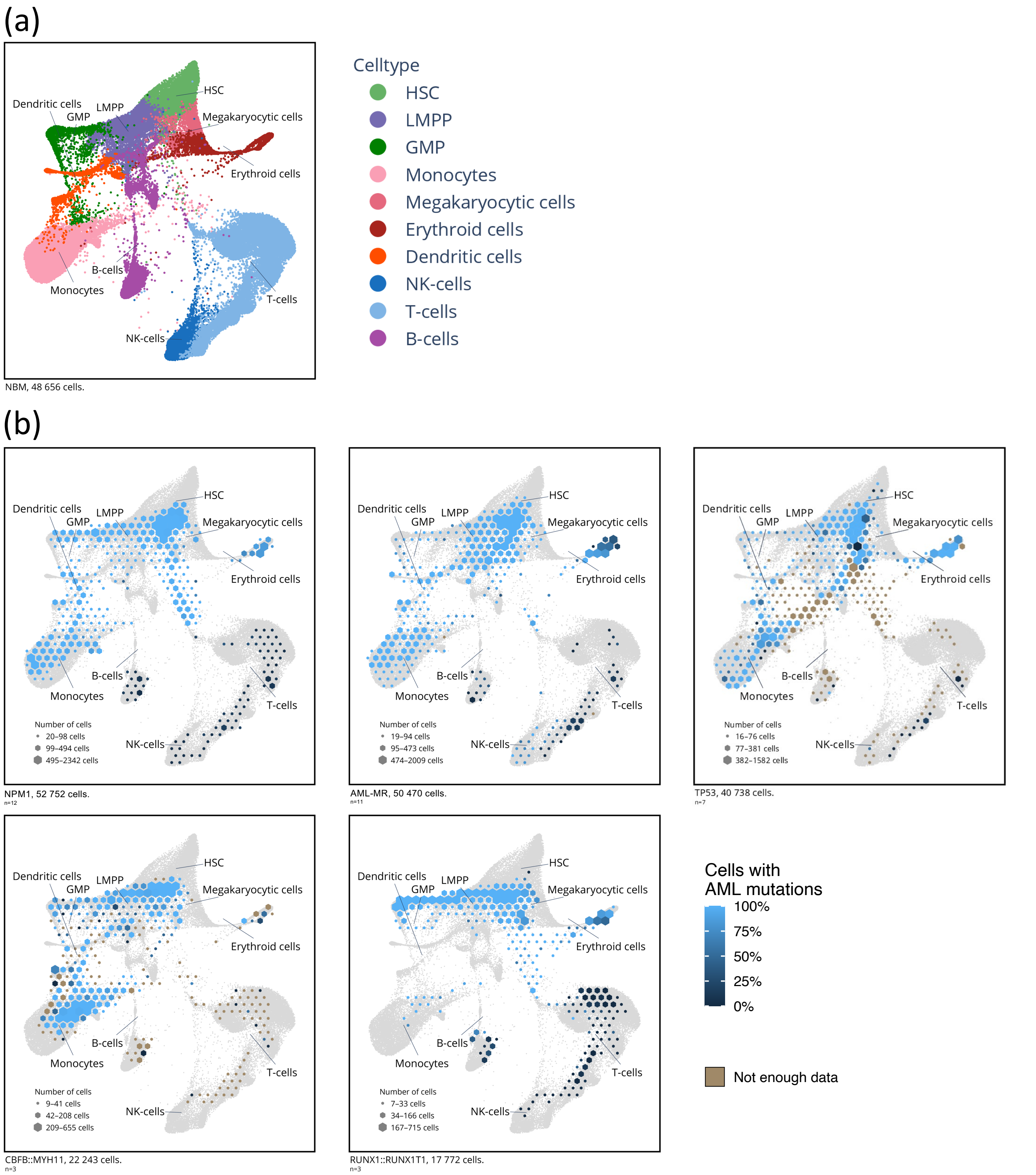

Supplementary Fig. 6 | Single cell projection of AML subtypes onto NBM reference knn force plot. (a) Knn force graph constructed from 48,656 normal bone marrow (NBM) cells from eight NBM samples. **(b)** Projection of single cells onto the reference NBM knn force graph (indicated in gray) for the subtypes *NPM1* (52,752 cells from 12 samples), AML-MR (50,470 cells from 11 samples), *TP53* (40,738 cells from 7 samples), *CBFB::MYH11* (22,243 cells from 3 samples), and *RUNX1::RUNX1T1* (17,772 cells from 3 samples). The number of cells projected onto a region is indicated by the size of each pixel and the proportion of mutated cells in that pixel is indicated by color. Pixels with too few genotype reads are marked in brown (three or fewer reads). The genomic subtype of the included samples, the number of represented cells, and the number of included samples (n) is indicated below each plot.

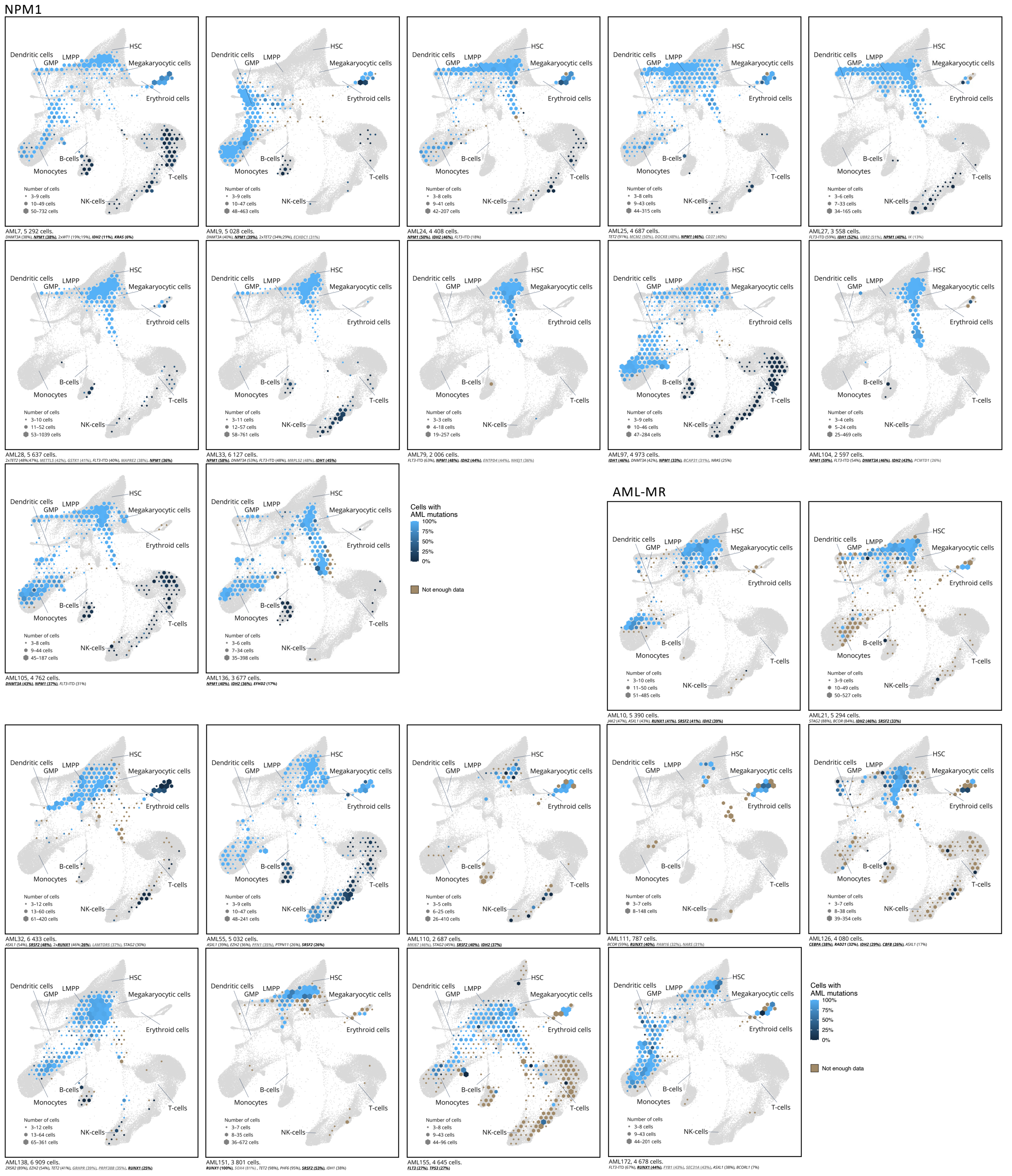

Supplementary Fig. 7 | Single cell projection of individual AML samples from NPM1 and AML-MR subtypes onto NBM reference knn force plot. Projection of single cells from AML samples onto the reference NBM knn force graph (indicated in gray), divided by sample. The number of cells projected onto a region is indicated by the size of each pixel and the proportion of mutated cells in that pixel is indicated by color. Pixels with too few genotype reads are marked in brown (three or fewer reads). Each plot represents a single sample, with sample identifier and number of cells indicated below the plot. A selection of genes mutated in each sample is presented below each plot, with the variant allele frequency indicated by WES denoted in parentheses. Genes targeted by scRNAmut-seq are indicated in bold. Genes with heterozygous mutations (used for inferring the proportion of mutated cells) are underlined. Genes with presumed passenger mutations are denoted in gray.

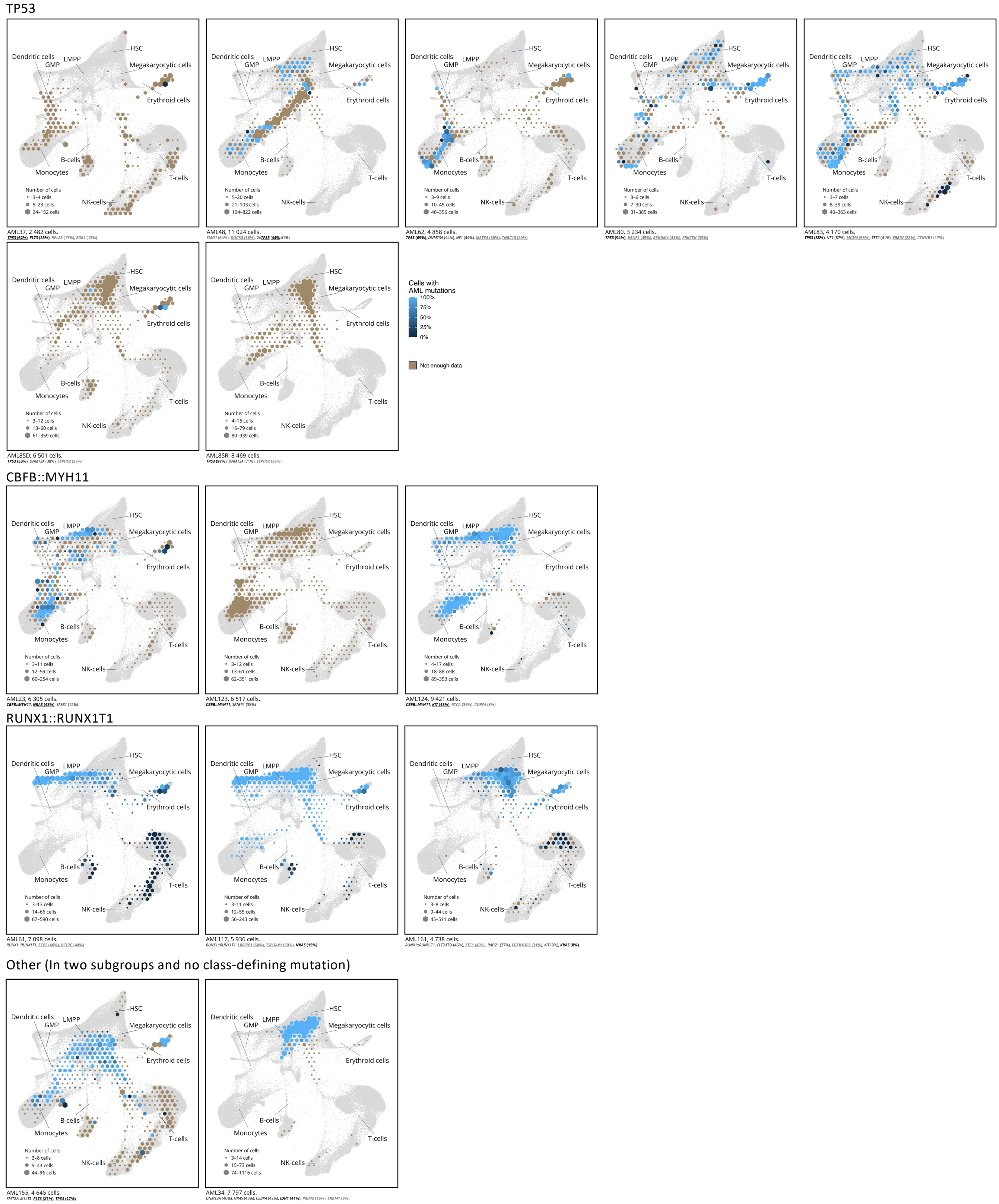

Supplementary Fig. 8 | Single cell projection of individual AML samples from TP53, CBFB::MYH11, RUNX1::RUNX1T1 and other subtypes onto NBM reference knn force plot. Projection of single cells from AML samples onto the reference NBM knn force graph (indicated in gray), divided by sample. The number of cells projected onto a region is indicated by the size of each pixel and the proportion of mutated cells in that pixel is indicated by color. Pixels with too few genotype reads are marked in brown (three or fewer reads). Each plot represents a single sample, with sample identifier and number of cells indicated below the plot. A selection of genes mutated in each sample is presented below each plot, with variant allele frequency from WES denoted in parentheses. Genes targeted by scRNAmut-seq are indicated in bold. Genes with heterozygous mutations (used for inferring the proportion of mutated cells) are underlined. Genes with presumed passenger mutations are denoted in gray.

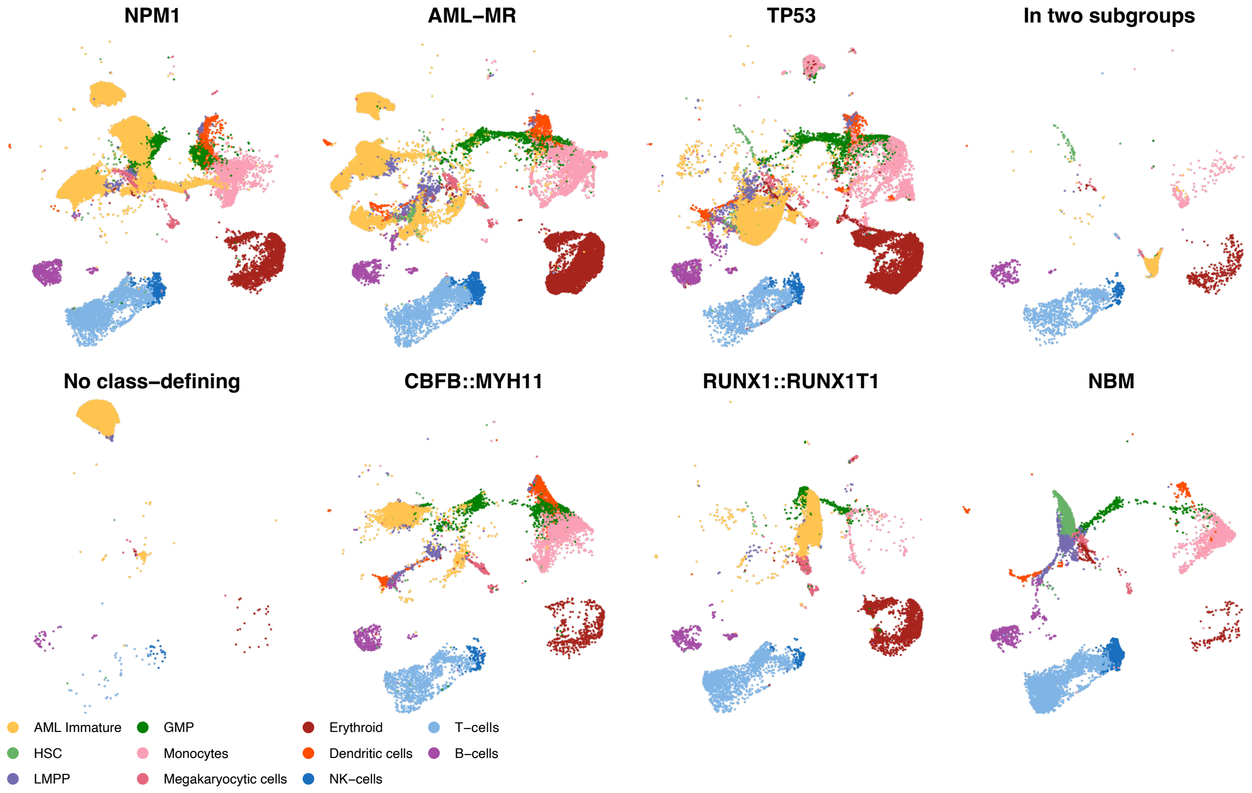

Supplementary Fig. 9 | AML immature cells in AML subtypes visualized using UMAP. UMAP representations of 245,073 cells from 38 AML samples and 8 NBM samples, separated into groups defined by AML subtype or NBM status. AML immature cells are indicated in yellow. Source data are provided as a Source Data file.

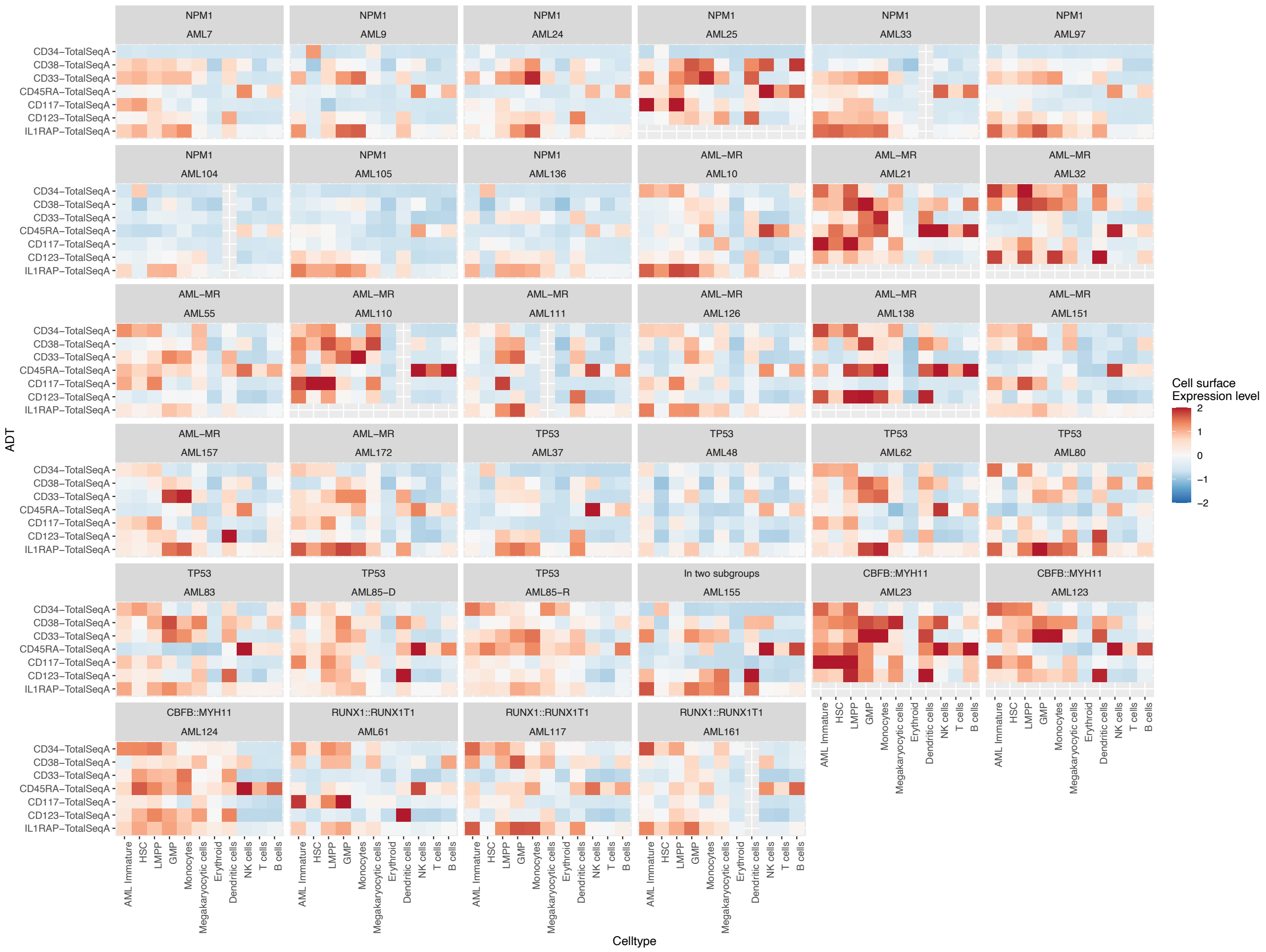

Supplementary Fig. 10 | Immunophenotypic features of distinct cell types in 34 AML samples as determined by scADT-seq. Heatmaps displaying the average cell surface expression of seven LSC markers for each cell type in 34 AML samples (scADT-seq data was not available from four AML samples; data for IL1RAP was only available from 27 AML samples). The AML immature cell type has an immunophenotype compatible with harboring LSC (i.e. CD34^{+}CD38$^{-/low}$ for CD34-positive AMLs and distinct expression profiles of CD33, CD45RA, CD117, CD123, and IL1RAP). Source data are provided as a Source Data file.

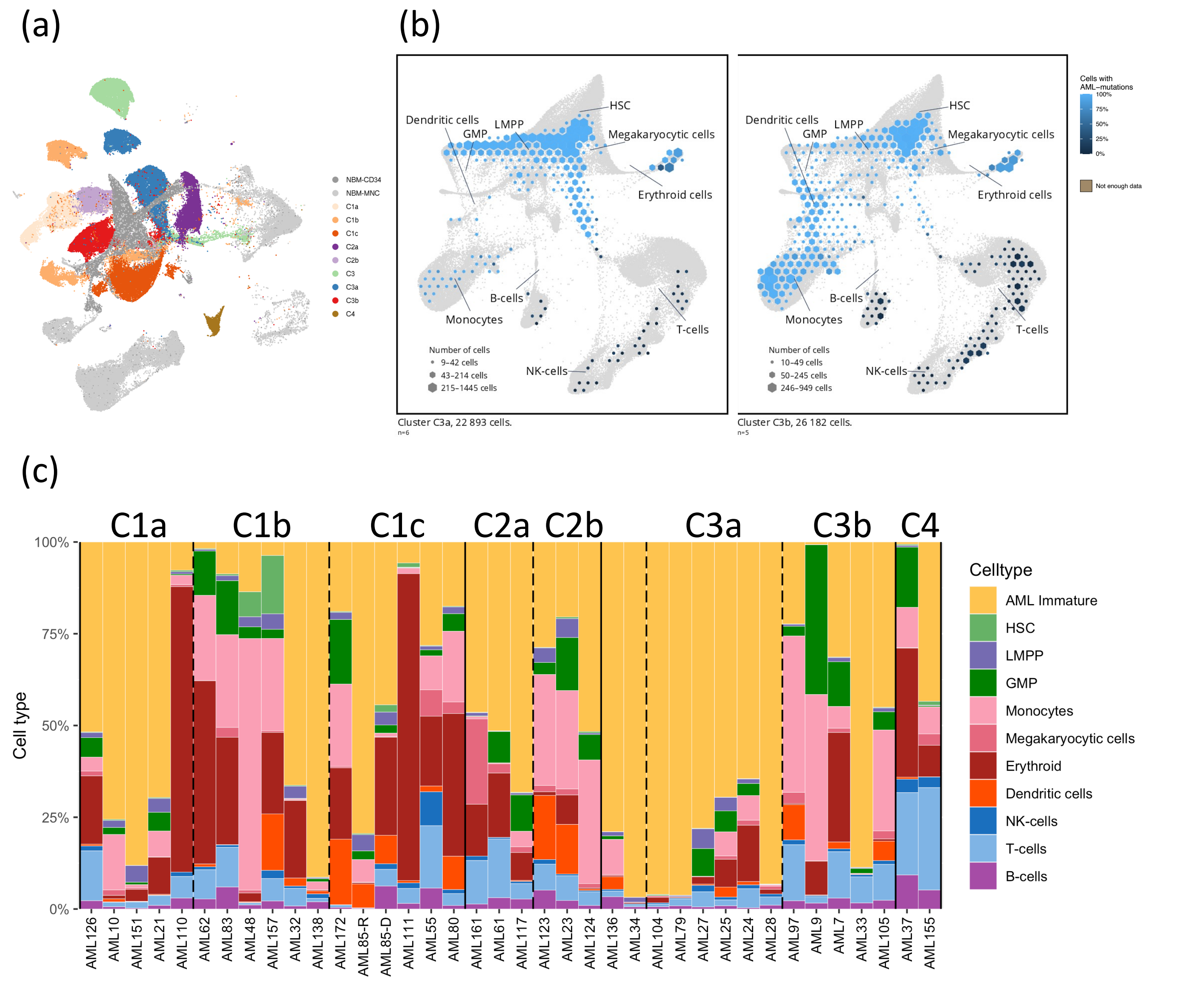

Supplementary Fig. 11 | UMAP clustering and cell type distribution of AML immature clusters from hierarchical clustering analysis. (a) UMAP representation of 245,073 cells from 38 AML samples and 8 NBM samples. Cells are colored by cluster identity of the AML sample based on hierarchical clustering on average gene expression of the AML immature cells from each AML sample (see Figure 3a). **(b)** Projection of single cells onto the reference NBM knn force graph (indicated in gray) from AML samples belonging to clusters C3a (22,893 cells from 6 samples) and C3b (26,182 cells from 5 samples), separated by cluster identity. Samples in cluster C3a (left) contain a high proportion of immature cells whereas samples from cluster C3b (right) contain a higher proportion of T-cells and differentiated AML cells. Number of projected cells and the number of included samples (n) is indicated below each plot. **(c)** Cell type distribution in samples from each cluster defined by hierarchical clustering on average gene expression of the AML immature cells from each AML sample (Figure 3a). Samples from cluster C3a contain a high proportion of AML immature cells whereas samples from cluster C3b contain a high proportion of GMPs, monocytes, and T-cells. Source data are provided as a Source Data file.

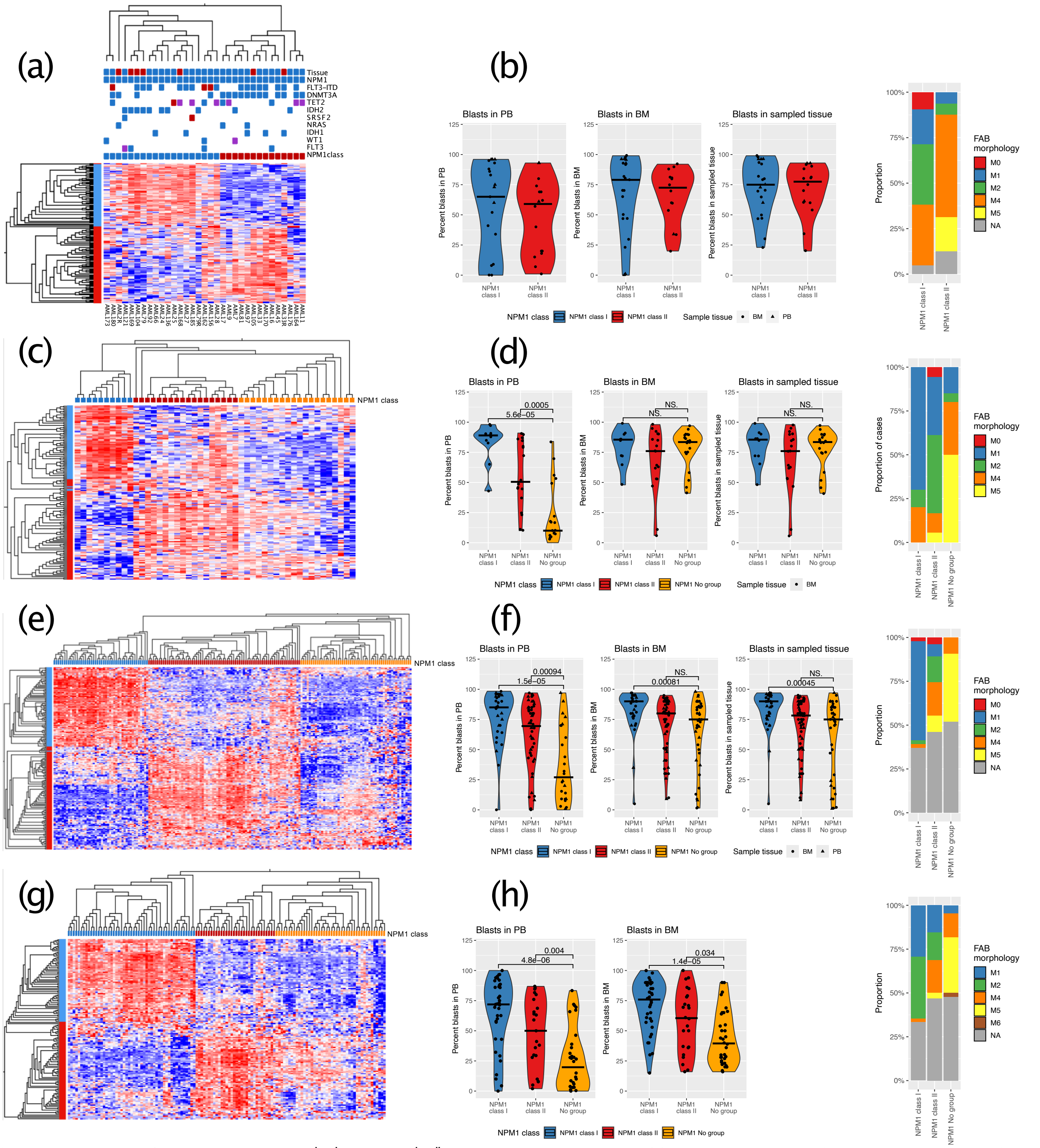

Supplementary Fig. 12 | Identification of *NPM1*$^{\text{class I}}$ and *NPM1*$^{\text{class II}}$ in bulk RNA sequencing data. (a) Hierarchical clustering of bulk gene expression data from 33 *NPM1*-mutated AML samples from the Lund dataset based on the expression of 180 genes differentially expressed between AML immature cells from *NPM1*$^{\text{class I}}$ (genes indicated in blue) and *NPM1*$^{\text{class II}}$ samples (genes indicated in red). Sample tissue and mutations in selected genes are indicated above each sample. In this dataset, 19 had an expression profile matching *NPM1*$^{\text{class I}}$ (samples indicated in blue) and 14 samples had an expression profile matching *NPM1*$^{\text{class II}}$ (samples indicated in red). **(b)** Percent blasts in PB, BM, and sample tissue (either PB or BM) for *NPM1*$^{\text{class I}}$ (n=19 samples) and *NPM1*$^{\text{class II}}$ cases (n=14 samples) from Lund (left side). FAB morphology for *NPM1*$^{\text{class I}}$ and *NPM1*$^{\text{class II}}$ cases from Lund (right side). **(c)** Hierarchical clustering of bulk gene expression data from 48 *NPM1*-mutated AML samples from the TCGA dataset[1] based on the expression of 180 genes differentially expressed between *NPM1*$^{\text{class I}}$ and *NPM1*$^{\text{class II}}$. **(d)** Percent blasts in PB, BM, and sample tissue (either PB or BM) for *NPM1*$^{\text{class I}}$ (n=10 samples), *NPM1*$^{\text{class II}}$ (n=18 samples), and unclassified cases (n=20 samples) from TCGA, with significant differences indicated by p-values (two-sided Mann-Whitney U test; left side). FAB morphology for *NPM1*$^{\text{class I}}$, *NPM1*$^{\text{class II}}$, and unclassified cases from TCGA (right side). **(e)** Hierarchical clustering of bulk gene expression data from 174 *NPM1*-mutated AML samples from the Beat-AML 2.0 dataset[5] based on the expression of 180 genes differentially expressed between *NPM1*$^{\text{class I}}$ and *NPM1*$^{\text{class II}}$. **(f)** Percent blasts in PB, BM, and sample tissue (either PB or BM) for *NPM1*$^{\text{class I}}$ (n=46 samples), *NPM1*$^{\text{class II}}$ (n=74 samples), and unclassified cases (n=54 samples) from Beat-AML 2.0, with significant differences indicated by p-values (two-sided Mann-Whitney U test; left side). FAB morphology for *NPM1*$^{\text{class I}}$, *NPM1*$^{\text{class II}}$, and unclassified cases from Beat-AML 2.0 (right side). **(g)** Hierarchical clustering of bulk gene expression data from 127 *NPM1*-mutated AML samples from the Clinseq dataset[6] based on the expression of 180 genes differentially expressed between *NPM1*$^{\text{class I}}$ and *NPM1*$^{\text{class II}}$. **(h)** Percent blasts in PB, BM, and sample tissue (either PB or BM) for *NPM1*$^{\text{class I}}$ (n=51 samples), *NPM1*$^{\text{class II}}$ (n=32 samples), and unclassified cases (n=44 samples) from Clinseq, with significant differences indicated by p-values (two-sided Mann-Whitney U test; left side). FAB morphology for *NPM1*$^{\text{class I}}$, *NPM1*$^{\text{class II}}$, and unclassified cases from Clinseq (right side). Source data are provided as a Source Data file.

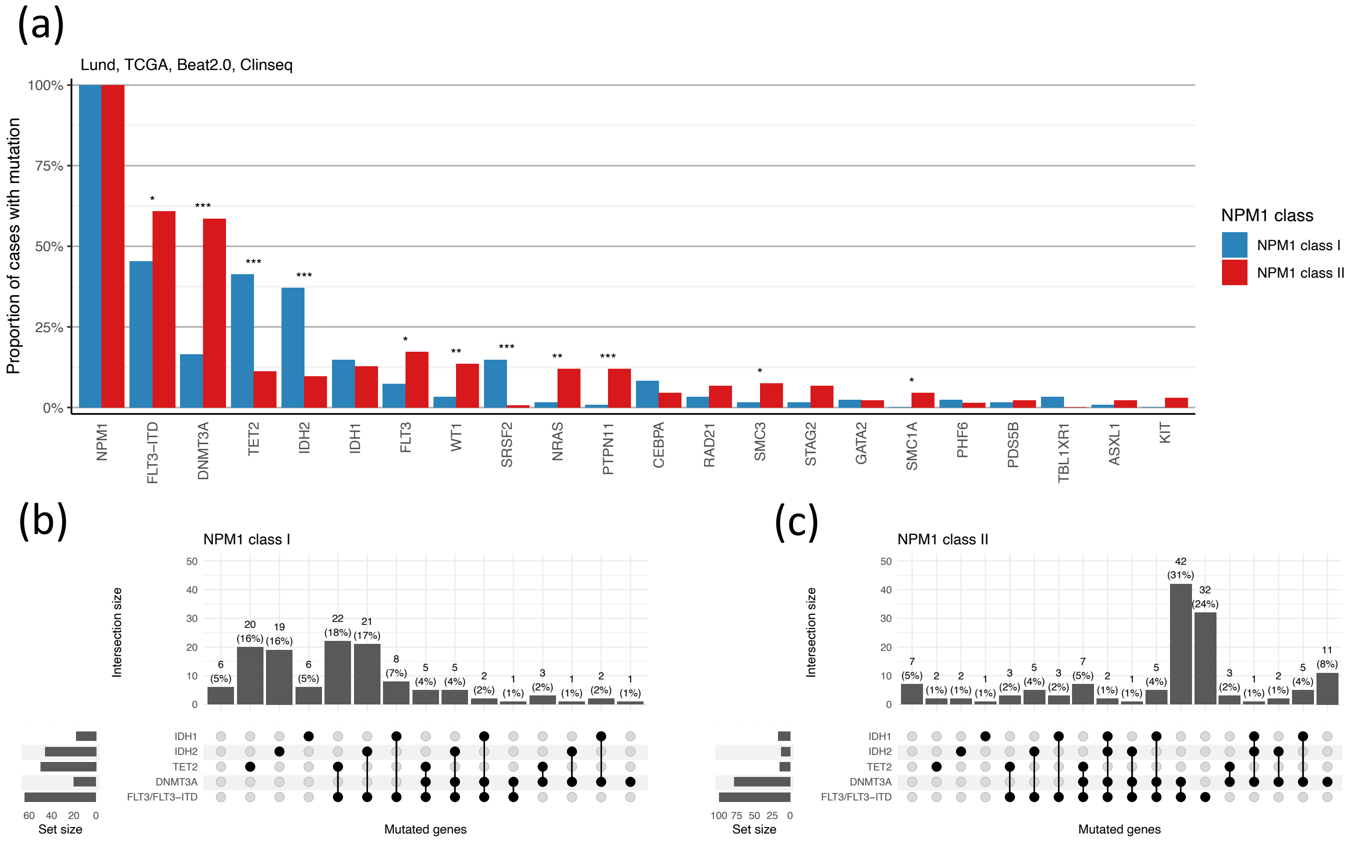

Supplementary Fig. 13 | Mutational patterns of *NPM1*$^{class\ I}$ and *NPM1*$^{class\ II}$ subtypes. (a) Proportion of cases with mutations in the most commonly mutated genes in the *NPM1*$^{class\ I}$ and *NPM1*$^{class\ II}$ subtypes**.** Significant differences between the groups are indicated with asterisks (*: p<0.05; **: p<0.01; ***: p<0.001; two-sided Fisher's exact test; n=121 *NPM1*$^{class\ I}$ samples and n=133 *NPM1*$^{class\ II}$ samples. Exact p values are provided in Source Data file.) **(b)** Frequency of co-mutational patterns in *NPM1*$^{class\ I}$ for the five most commonly mutated genes in AML with *NPM1*-mutations overall. **(c)** Frequency of co-mutational patterns in *NPM1*$^{class\ II}$ for the four most commonly mutated genes in AML with *NPM1*-mutations overall. Source data are provided as a Source Data file.

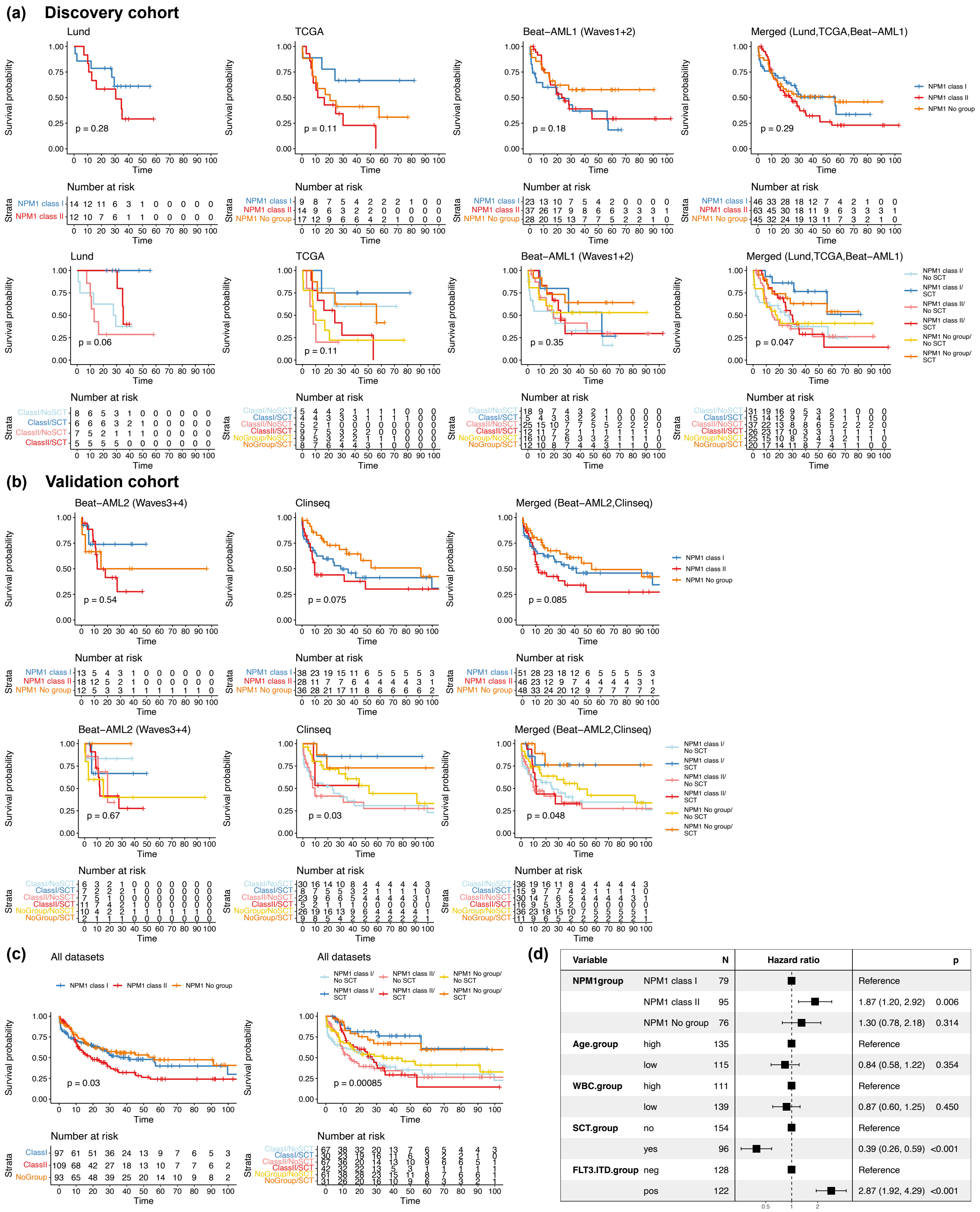

Supplementary Fig. 14 | Overall survival for *NPM1*$^{class\ I}$ and *NPM1*$^{class\ II}$ subtypes. (a) Top row: Overall survival for all patients with *NPM1* mutated AML that were treated with curative intent in the discovery cohort (Lund, TCGA, and Beat-AML (waves1+2) studies, n=154 patients) shown as Kaplan-Meier curves for each dataset separately and merged together. Bottom row: Overall survival for all patients with *NPM1* mutated AML that were treated with curative intent in the discovery cohort, as above, but with separate curves for patients that received and did not receive a hematopoietic stem cell transplantation (p-values from two-sided Logrank test). **(b)** Top row: Overall survival for all patients with *NPM1* mutated AML that were treated with curative intent in the validation cohort (Beat-AML (waves3+4) and Clinseq studies; n=145 patients) shown as Kaplan-Meier curves for each dataset separately and merged together. Bottom row: Overall survival for all patients with *NPM1* mutated AML that were treated with curative intent in the validation cohort, as above, but with separate curves for patients that received and did not receive a hematopoietic stem cell transplantation (p-values from two-sided Logrank test). **(c)** Overall survival for all patients with *NPM1* mutated AML that were treated with curative intent in all the datasets merged (including both discovery and validation cohorts, n=299 patients) shown as Kaplan-Meier curves based on *NPM1* class (left) or both *NPM1* class and hematopoietic stem cell transplantation status (right; p-values from two-sided Logrank test) **(d)** Forest plot illustrating the hazard ratio from a multivariate Cox proportional hazard model of the overall survival for all datasets merged. The following covariates are included: NPM1group (*NPM1*$^{class\ I}$, *NPM1*$^{class\ II}$, *NPM1* No group), Age.group (high: >= 60 years, low: < 60 years), WBC.group (high: >$42x10^9$/L; low: <= $42x10^9$/L), SCT.group (no: no stem cell transplantation; yes: received stem cell transplantation), and FLT3.ITD.group (neg: negative for *FLT3*-ITD; pos: positive for *FLT3*-ITD). Source data are provided as a Source Data file.

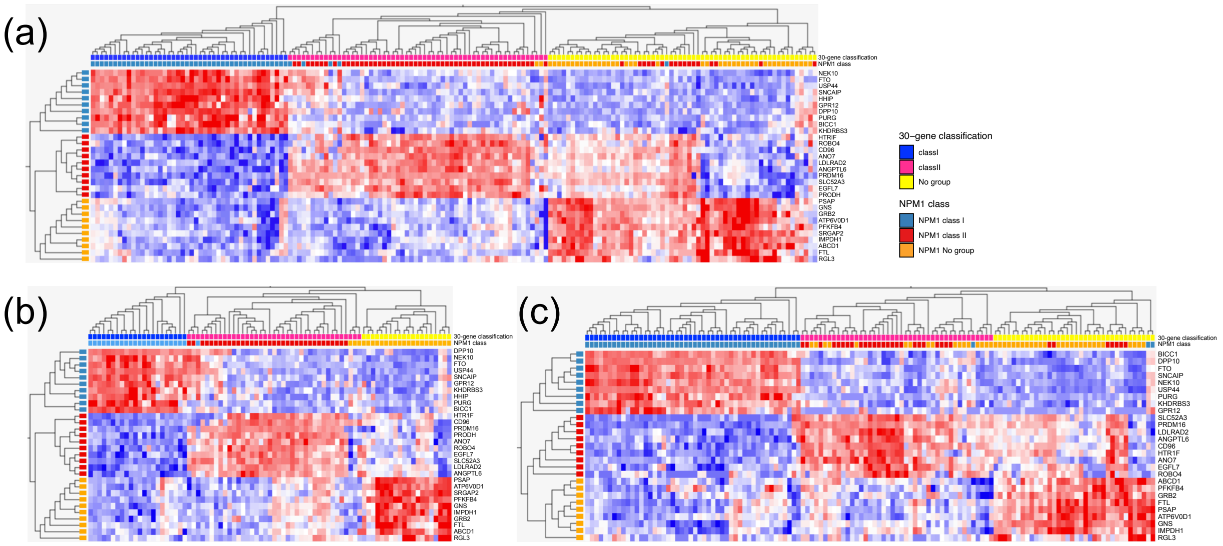

Supplementary Fig. 15 | Identification of *NPM1*$^{\text{class I}}$ and *NPM1*$^{\text{class II}}$ in bulk RNA sequencing data using a reduced list of 30 genes. (a) Hierarchical clustering of bulk gene expression data from 162 *NPM1*-mutated AML samples from the Lund, TCGA, and Beat-AML (before 2018) datasets based on the expression of 30 differentially expressed genes. Differentially expressed genes are indicated by gene symbol to the right and by *NPM1* class to the left (upregulated in *NPM1*$^{\text{class I}}$ in blue, *NPM1*$^{\text{class II}}$ in red, and in unclassified cases in orange). The classification of each case according to the expression of 30 genes and the original classification based on 180 genes is indicated above the heatmap. In total, 24 cases are classified differently using the reduced list. Twenty of the misclassified cases go to or from "*NPM1* No group". **(b)** Hierarchical clustering of bulk gene expression data from 81 *NPM1*-mutated AML samples from the Beat-AML dataset (after 2018) based on the expression of 30 differentially expressed genes. Differentially expressed genes are indicated by gene symbol to the right and by *NPM1* class to the left (upregulated in *NPM1*$^{\text{class I}}$ in blue, *NPM1*$^{\text{class II}}$ in red, and in unclassified cases in orange). The classification of each case according to the expression of 30 genes and the original classification based on 180 genes is indicated above the heatmap. In total, four cases are classified differently using the reduced list. Three of the misclassified cases go from "*NPM1* No group" to *NPM1*$^{\text{class II}}$. **(c)** Hierarchical clustering of bulk gene expression data from 127 *NPM1*-mutated AML samples from the Clinseq dataset based on the expression of 30 differentially expressed genes. Differentially expressed genes are indicated by gene symbol to the right and by *NPM1* class to the left (upregulated in *NPM1*$^{\text{class I}}$ in blue, *NPM1*$^{\text{class II}}$ in red, and in unclassified cases in orange). The classification of each case according to the expression of 30 genes and the original classification based on 180 genes is indicated above the heatmap. In total, 27 cases are classified differently using the reduced list. Twenty-six of the misclassified cases go to or from "*NPM1* No group".

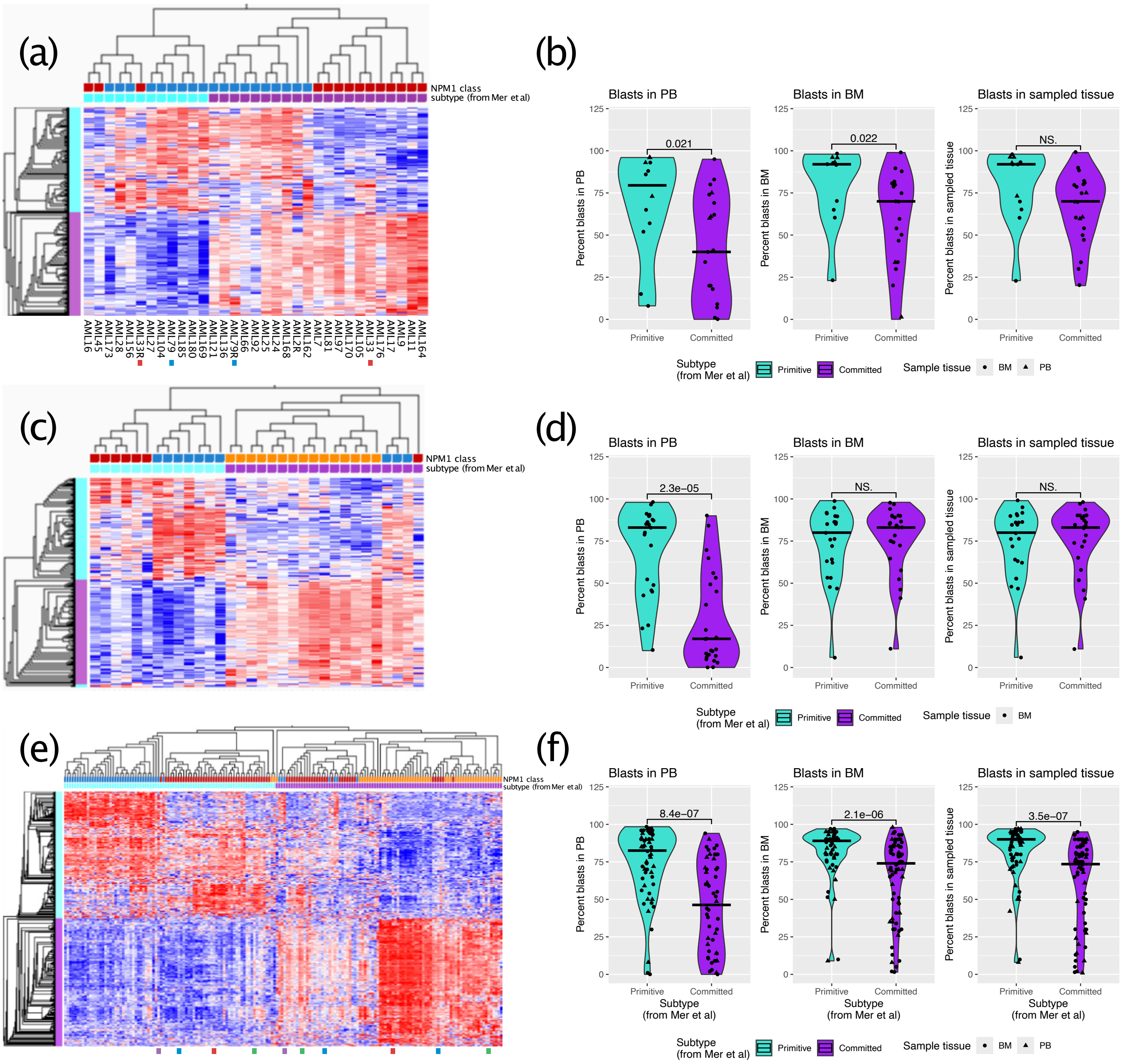

Supplementary Fig. 16 | Primitive and committed subtypes described by Mer et. al does not overlap with *NPM1*$^{\text{class I}}$ and *NPM1*$^{\text{class II}}$ subtypes. (a) Hierarchical clustering of bulk gene expression data from 33 *NPM1*-mutated AML samples from the Lund dataset based on the expression of the 100 most highly expressed genes in the primitive subtype (genes indicated in turquoise) and the 100 most highly expressed genes in the committed subtype (genes indicated in purple), as described by Mer et al.[7] *NPM1*$^{\text{class I}}$ (blue)/*NPM1*$^{\text{class II}}$ (red) classification and primitive (turquoise)/committed (purple) classification (based on this hierarchical clustering) is indicated above the heatmap. Two diagnose-relapse pairs that switch between primitive and committed subtypes are indicated below the heatmap in red and blue, respectively. **(b)** Percent blasts in PB, BM, and sample tissue (either PB or BM) for primitive (n=12 samples) and committed cases (n=21 samples) from Lund, with significant differences indicated by p-values (two-sided Mann-Whitney U test). **(c)** Hierarchical clustering of bulk gene expression data from 48 *NPM1*-mutated AML samples from the TCGA dataset[1] based on the expression of the 100 most highly expressed genes in the primitive subtype (genes indicated in turquoise) and the 100 most highly expressed genes in the committed subtype (genes indicated in purple), as described by Mer et al.[7] *NPM1*$^{\text{class I}}$ (blue)/*NPM1*$^{\text{class II}}$ (red) classification and primitive (turquoise)/committed (purple) classification (based on this hierarchical clustering) is indicated above the heatmap. **(d)** Percent blasts in PB, BM, and sample tissue (either PB or BM) for primitive (n=23 samples) and committed cases (n=25 samples) from TCGA, with significant differences indicated by p-values (two-sided Mann-Whitney U test). **(e)** Hierarchical clustering of bulk gene expression data from 174 *NPM1*-mutated AML samples from the Beat-AML 2.0 dataset[5] based on the expression of the 300 most highly expressed genes in the primitive subtype (genes indicated in turquoise) and the 300 most highly expressed genes in the committed subtype (genes indicated in purple), specifically in the Beat-AML cohort as described by Mer et al.[7] *NPM1*$^{\text{class I}}$ (blue)/*NPM1*$^{\text{class II}}$ (red) classification and primitive (turquoise)/committed (purple) classification (based on this hierarchical clustering) is indicated above the heatmap. Samples from four AMLs that switch between primitive and committed subtypes between sampling timepoints are indicated below the heatmap with red, blue, green and purple boxes. **(f)** Percent blasts in PB, BM, and sample tissue (either PB or BM) for primitive (n=84 samples) and committed cases (n=90 samples) from Beat-AML 2.0, with significant differences indicated by asterisks (two-sided Mann-Whitney U test). Source data are provided as a Source Data file.

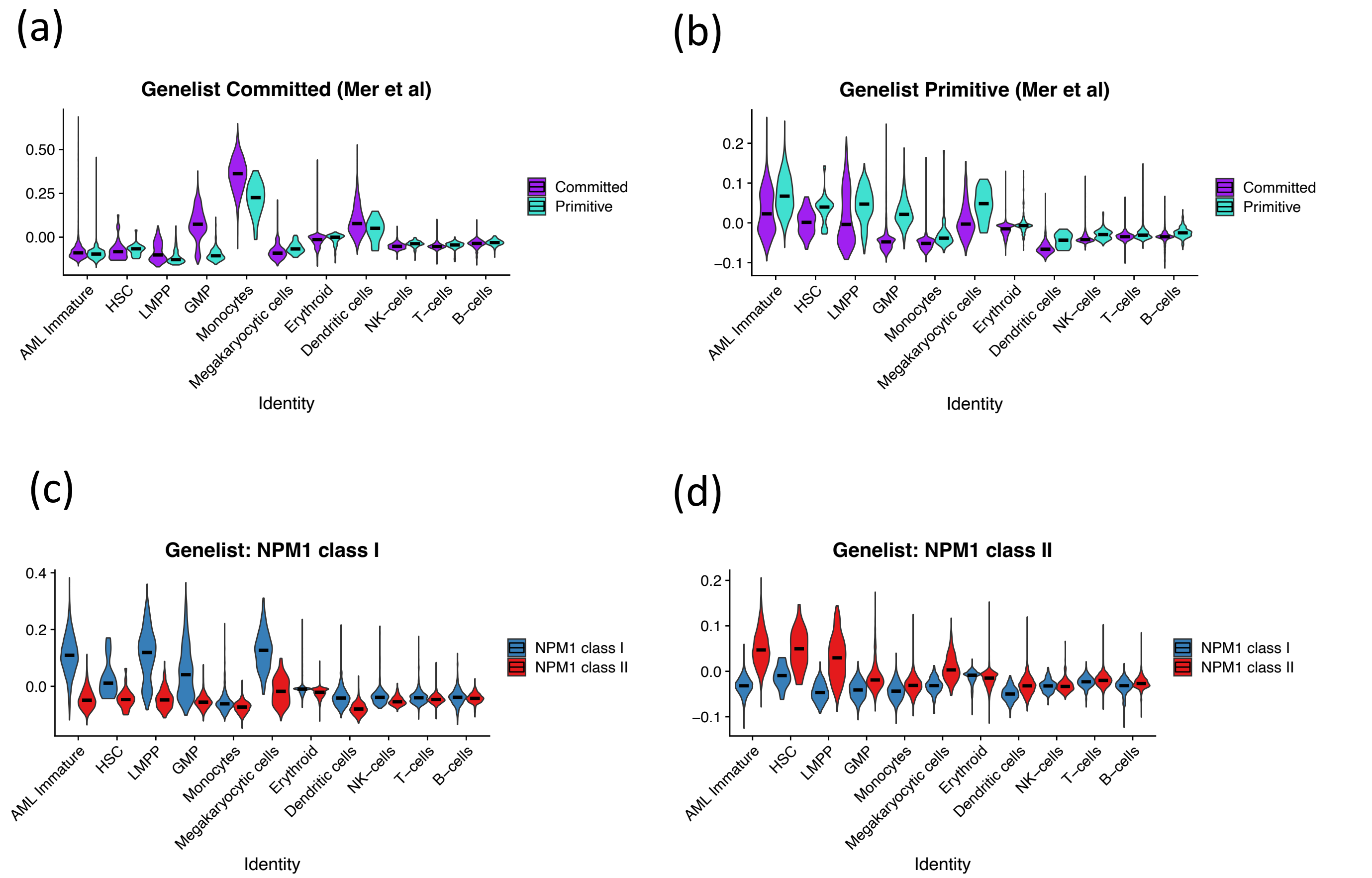

Supplementary Fig. 17 | Gene expression across cell types for genes associated with *NPM1* subtypes. (a) Average expression of genes defining the committed subtype across cell types in single cell data from twelve *NPM1*-mutated AML samples, divided into committed and primitive samples. The gene expression module is defined by the top 100 overexpressed genes in the committed subtype, as described by Mer et al.[7] **(b)** Average expression of genes defining the primitive subtype across cell types in single cell data from twelve *NPM1*-mutated AML samples, divided into committed and primitive samples. The gene expression module is defined by the top 100 overexpressed genes in the primitive subtype, as described by Mer et al.[7] **(c)** Average expression of genes defining $NPM1^{\text{class I}}$ across cell types in single cell data from *NPM1*-mutated AML samples, divided into $NPM1^{\text{class I}}$ and $NPM1^{\text{class II}}$. The gene expression module is defined by 79 genes specifically expressed in immature cells in $NPM1^{\text{class I}}$ samples. **(d)** Average expression of genes defining $NPM1^{\text{class II}}$ across cell types in single cell data from *NPM1*-mutated AML samples, divided into $NPM1^{\text{class I}}$ and $NPM1^{\text{class II}}$. The gene expression module is defined by 101 genes specifically expressed in immature cells in $NPM1^{\text{class II}}$ samples. Source data are provided as a Source Data file.

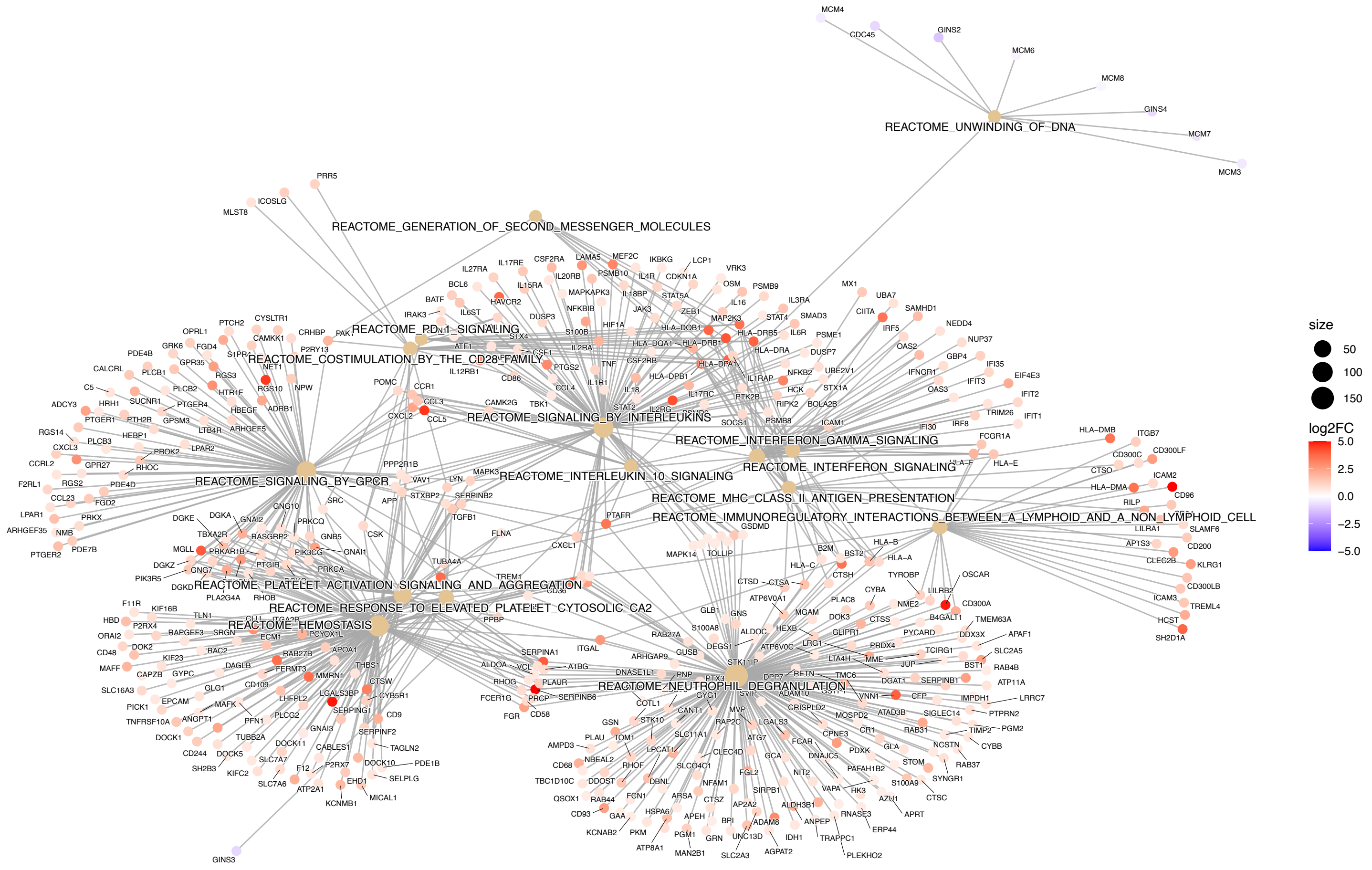

Supplementary Fig. 18 | Gene set enrichment analysis between *NPM1*$^{class\ I}$ and *NPM1*$^{class\ II}$ subtypes. Network plot visualizing gene set enrichment analysis for differentially expressed genes between AML immature cells in *NPM1*$^{class\ I}$ and *NPM1*$^{class\ II}$ samples. The fifteen most enriched of the msigdb canonical pathways reactome gene sets are included, together with core enriched genes from these gene sets.

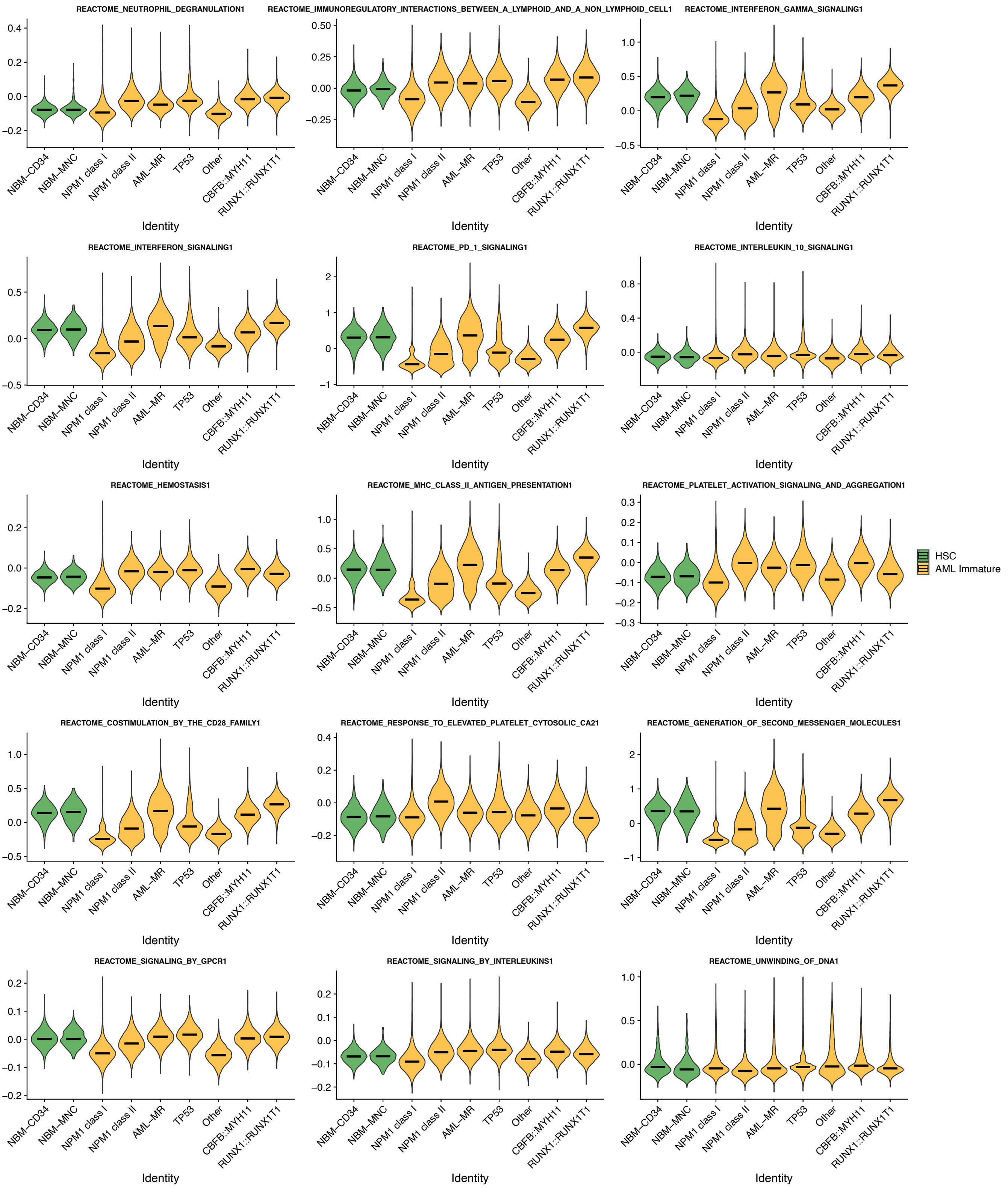

Supplementary Fig. 19 | Expression level of core expressed genes from GSEA enriched pathways in AML immature cells and NBM HSC cells. Expression level of modules defined by the core enriched genes from the fifteen most enriched gene sets from reactome is visualized in HSC cells from NBM-CD34 (n=5,958 cells) and NBM-MNC (n=282 cells) and in AML immature cells from the subtypes *NPM1* class I (n=21,458 cells), *NPM1* class II (n=10,382 cells), AML-MR (n=26,549 cells), *TP53* (n=12,142 cells), Other (n=9,565 cells), *CBFB::MYH11* (n=8,026 cells), and *RUNX1::RUNX1T1* (n=9,900 cells). Source data are provided as a Source Data file.

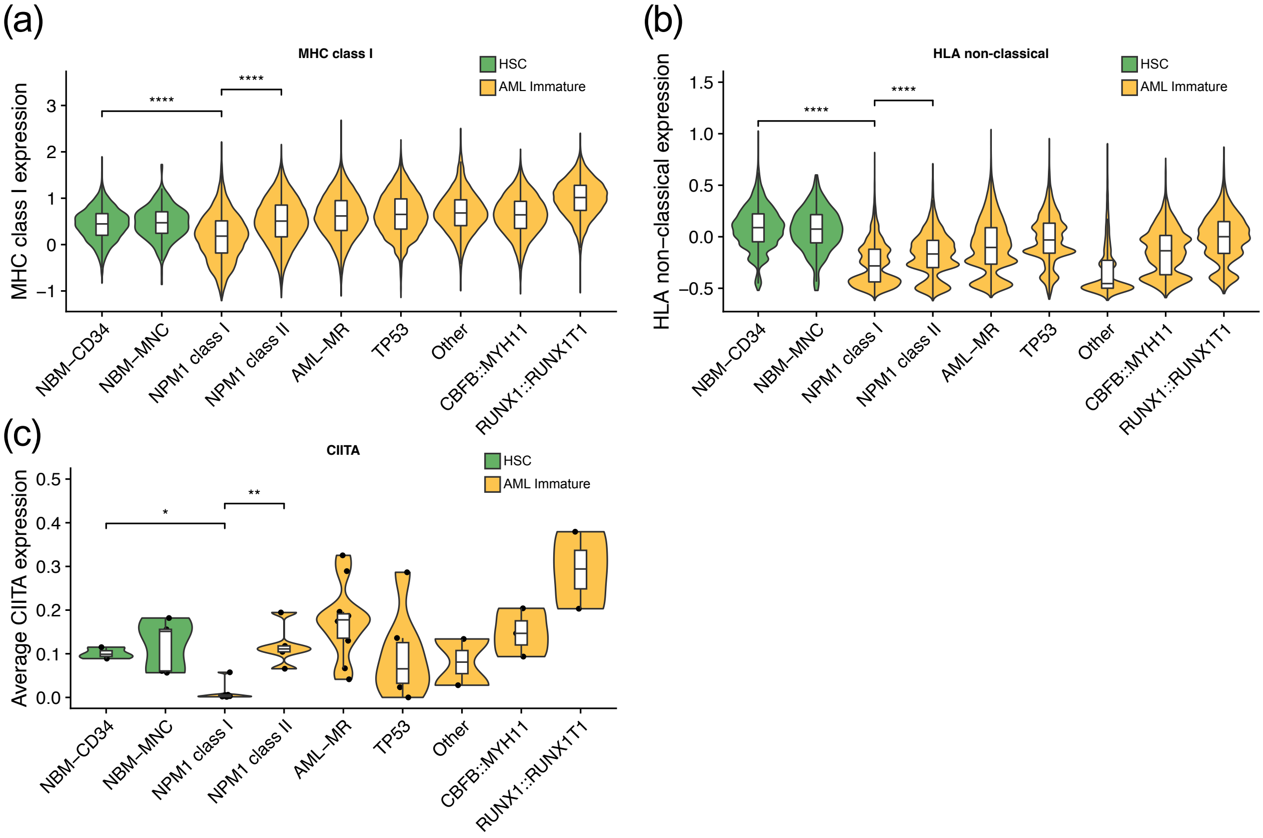

Supplementary Fig. 20 | Expression of MHC class I genes, non-classical HLA genes, and the transactivator CIITA in AML immature cells and NBM HSC cells. **(a)** Expression of MHC class I genes (HLA-A, HLA-B, HLA-C) in HSC cells from NBM-CD34 (n=5,958 cells) and NBM-MNC (n=282 cells) and in AML immature cells from the subtypes *NPM1* class I (n=21,458 cells), *NPM1* class II (n=10,382 cells), AML-MR (n=26,549 cells), *TP53* (n=12,142 cells), Other (n=9,565 cells), *CBFB::MYH11* (n=8,026 cells), and *RUNX1::RUNX1T1* (n=9,900 cells), based on scRNA-seq data. Box-and-whisker plots inside violin plots show median (center line), first and third quartiles (hinges), and 1.5x interquartile range (whiskers). The expression is significantly lower in AML immature cells from *NPM1* class I samples compared to both HSC cells from NBM-CD34 (two-sided Mann-Whitney U test, P<2.2e-16) and AML immature cells from *NPM1* class II samples (two-sided Mann-Whitney U test, P<2.2e-16). **(b)** Expression of HLA non-classical genes (HLA-E, HLA-F, HLA-G) in individual AML immature cells from AML samples and HSC cells from NBM samples, based on scRNA-seq data. Box-and-whisker plots inside violin plots show median (center line), first and third quartiles (hinges), and 1.5x interquartile range (whiskers). The expression is significantly lower in AML immature cells from *NPM1* class I samples compared to both HSC cells from NBM-CD34 (two-sided Mann-Whitney U test, P<2.2e-16) and AML immature cells from *NPM1* class II samples (two-sided Mann-Whitney U test, P<2.2e-16). **(c)** Average expression of CIITA for HSC cells from individual samples from NBM-CD34 (n=3 samples) and NBM-MNC (n=5 samples) and AML immature cells from individual samples from the subtypes *NPM1* class I (n=7 samples), *NPM1* class II (n=5 samples), AML-MR (n=11 samples), TP53 (n=7 samples), Other (n=2 samples), *CBFB::MYH11* (n=3 samples), and *RUNX1::RUNX1T1* (n=3 samples). Points indicate the average value for individual AML or NBM samples, box-and-whisker plots inside violin plots show median (center line), first and third quartiles (hinges), and 1.5x interquartile range (whiskers). The expression is significantly lower in AML immature cells from *NPM1* class I samples compared to both HSC cells from NBM-CD34 (two-sided Mann-Whitney U test, P=0.017) and AML immature cells from *NPM1* class II samples (two-sided Mann-Whitney U test, P=0.0025). Source data are provided as a Source Data file.

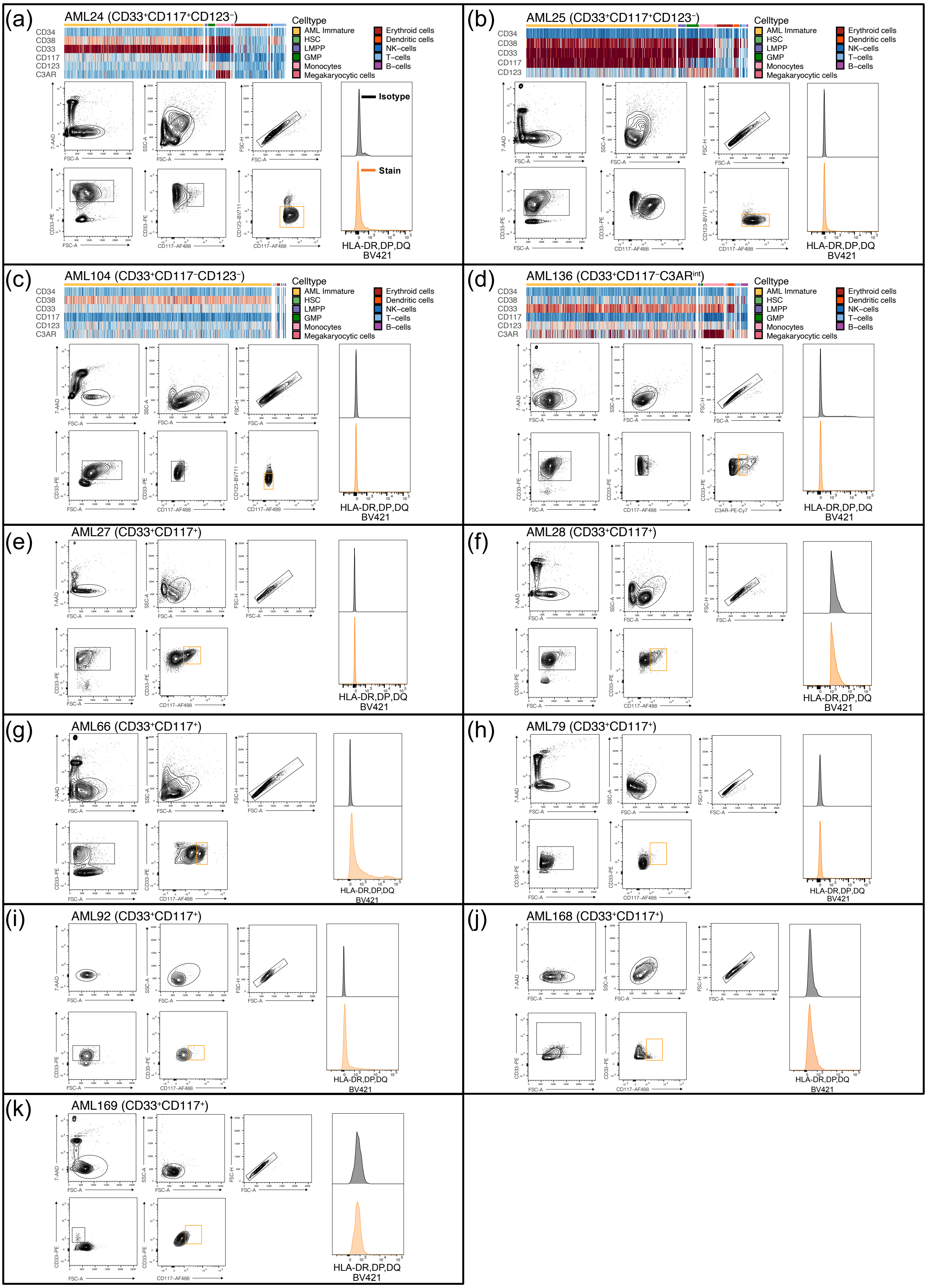

Supplementary Fig. 21 | Cell surface markers used to define AML immature cells in *NPM1*$^{\text{class I}}$ samples. (a-d) Heatmaps illustrating the expression of cell surface markers in AML24 (n=4,408 cells), AML25 (n=4,687 cells), AML104 (n=2,597 cells) and AML136 (n=3,677 cells) as identified by scADT-seq (top). Cell type is indicated by color above heatmap. Scatter plots showing the gating strategy to identify AML immature cells by flow cytometry (bottom left). Histograms showing HLA-DR, -DP, and -DQ expression in the AML immature cells (bottom right). **(e-k)** Scatter plots showing the gating strategy to identify AML immature cells in AML27, AML28, AML66, AML79, AML92, AML168, and AML169 by flow cytometry, defined as CD33^{+}CD117^{+} (left). Histograms showing HLA-DR, -DP, and -DQ expression in the AML immature cells of AML27, AML28, AML66, AML79, AML92, AML168, and AML169 (right).

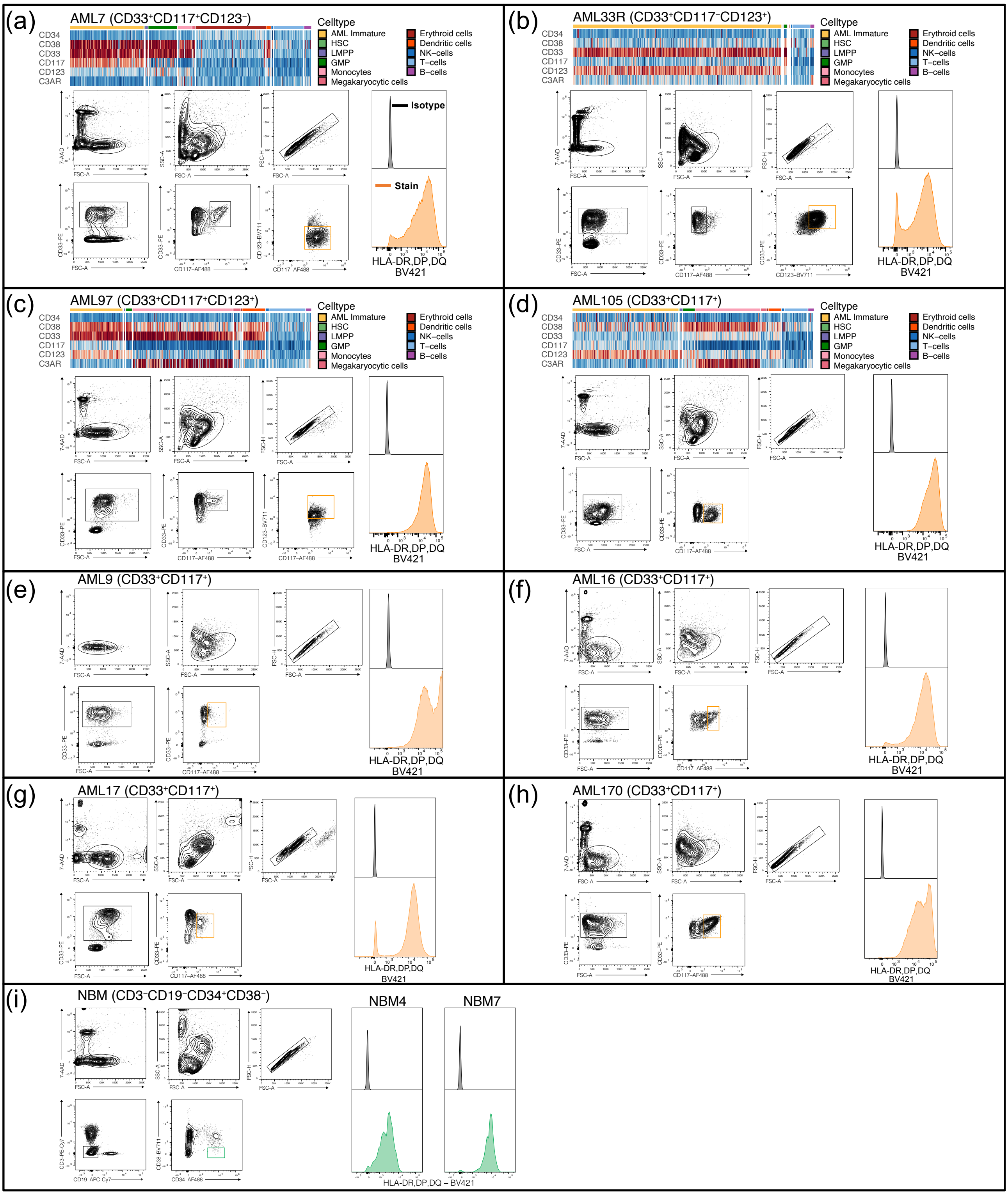

Supplementary Fig. 22 | Cell surface markers used to define AML immature cells in *NPM1*$^{\text{class II}}$ samples. (a-d) Heatmaps illustrating the expression of selected cell surface markers in AML7 (n=5,292 cells), AML33R (n=6,127 cells), AML97 (n=4,973 cells), and AML105 (n=4,762 cells) as identified by scADT-seq (top). Cell type is indicated by color above heatmap. Scatter plots showing the gating strategy to identify AML immature cells by flow cytometry using immune profiles defined by scADT-seq (bottom left). Histograms showing HLA-DR, -DP, and -DQ expression in the AML immature cells (bottom right). **(e-h)** Scatter plots showing the gating strategy to identify AML immature cells in AML9, AML16, AML17, and AML170, defined as CD33^{+}CD117^{+}, by flow cytometry (left). Histograms showing HLA-DR, -DP, and -DQ expression in the AML immature cells of AML9, AML16, AML17, and AML170 (right). **(i)** Scatter plots showing the gating strategy for identifying immature (CD34^{+}CD38^{-}) cells from NBM using flow cytometry. Histograms showing HLA-DR, -DP, and -DQ expression in CD34^{+}CD38^{-} cells from two NBM samples.

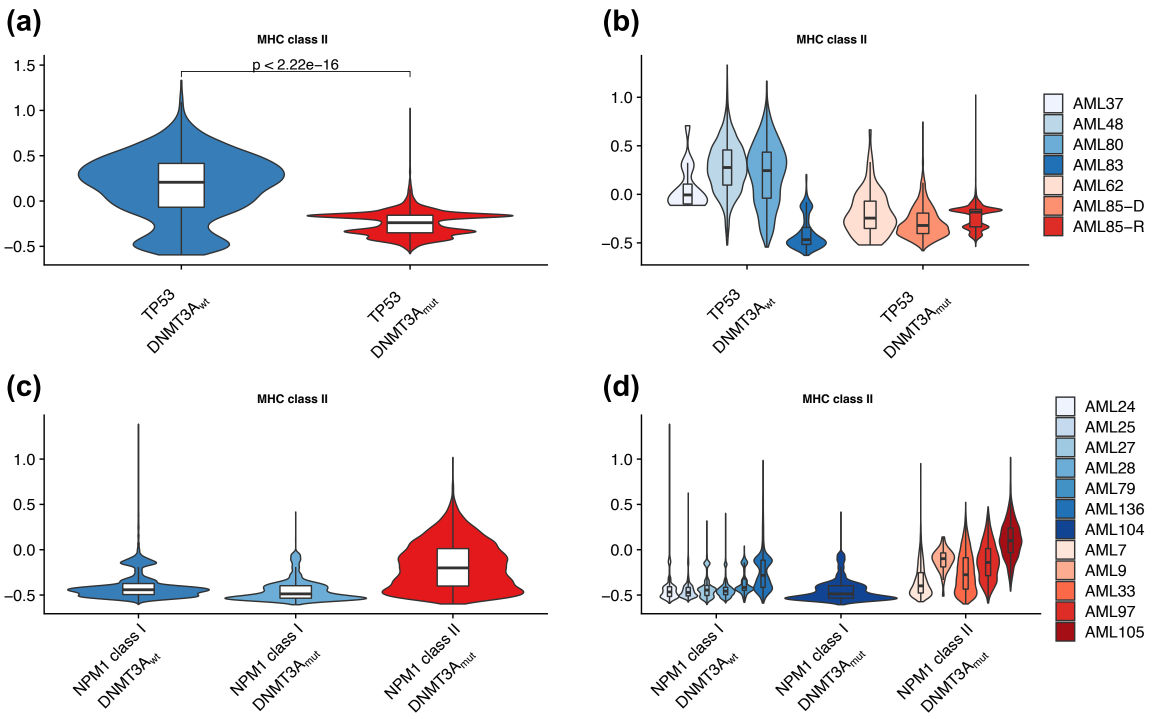

Supplementary Fig. 23 | Expression of genes encoding MHC class II components in AML immature cells from *TP53*-mutated and *NPM1*$^{\text{class I}}$/*NPM1*$^{\text{class II}}$ samples divided by *DNMT3A* mutation status. (a) Single cell expression of genes encoding MHC class II components in AML immature cells from *TP53*-mutated AMLs harboring wildtype *DNMT3A* (*TP53*/$DNMT3A_{wt}$, 4 cases, n=2,442 cells) compared with mutated DNMT3A (*TP53*/$DNMT3A_{mut}$, 3 cases, n=9,700 cells). The expression of genes encoding MHC class II components in this subtype is significantly higher in *DNMT3A* wild type cases. (two-sided Mann-Whitney U test) **(b)** Same data as in (a), presented individually for each sample (AM37: n=16 cells, AML48: n=1,497 cells, AML80: n=566 cells, AML83: n=363 cells, AML62: n=87 cells, AML85-D: n=2,885 cells, AML85-R: n=6,728 cells). **(c)** Single cell expression of genes encoding MHC class II components compared between AML immature cells from *NPM1*$^{\text{class I}}$/*DNMT3A* wildtype (6 cases, n=18,964 cells), *NPM1*$^{\text{class I}}$/*DNMT3A* mutated (1 case, n=2,494 cells), and *NPM1*$^{\text{class II}}$/*DNMT3A* mutated AMLs (5 cases, n=10,382 cells). The data set did not include any *NPM1*$^{\text{class II}}$/*DNMT3A* wildtype cases. **(d)** Same data as in (c) presented individually for each sample (AML24: n=2,842 cells, AML25: n=3,259 cells, AML27: n=2,776 cells, AML28: n=5,232 cells, AML79: n=1,931 cells, AML136: n=2,903 cells, AML104: n=2,494 cells, AML7: n=1,659 cells, AML9: n=36 cells, AML33: n=5,432 cells, AML97: n=1,111 cells, AML105: n=2,144 cells). The single *NPM1*$^{\text{class I}}$/*DNMT3A* mutated case exhibits expression of MHC class II components on the same level or below other *NPM1*$^{\text{class I}}$ AMLs, and clearly below the level of *NPM1*$^{\text{class II}}$ AMLs. The data from both *TP53*-mutated and *NPM1*-mutated AMLs indicate that high MHC class II expression (as observed in *NPM1*$^{\text{class II}}$ AMLs) is not a general feature of AML with mutated *DNMT3A*. Source data are provided as a Source Data file.

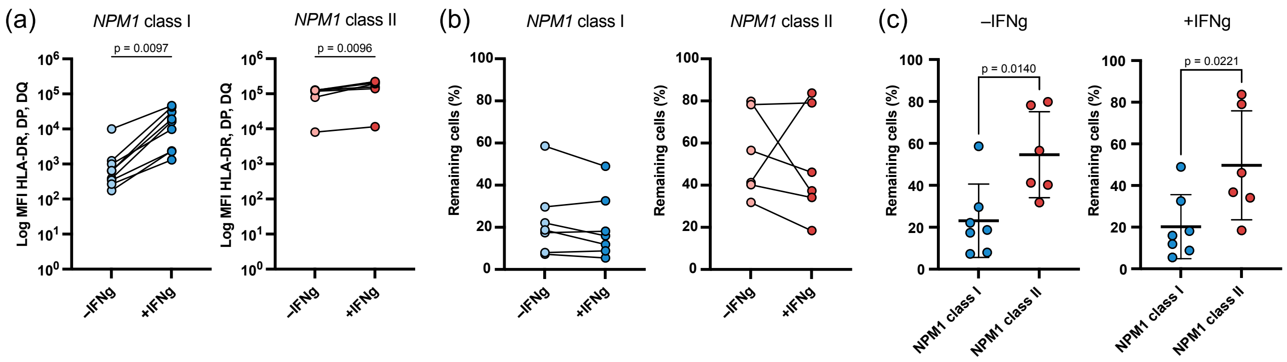

Supplementary Fig. 24 | AML cells treated with interferon gamma display upregulation of MHC class II surface molecules and increased T cell sensitivity. (a) MHC class II surface molecule expression, measured by FACS, on AML cells from *NPM1* class I (left panel; n=9 samples) and *NPM1* class II (right panel; n=7 samples) after three-day culture without interferon gamma (-IFNg) or with interferon gamma (+IFNg). MHC class II surface molecules were significantly upregulated with IFNg treatment on AML cells from both *NPM1* class I and *NPM1* class II (paired two-sided Mann-Whitney U test). **(b)** Survival of AML cells after three-day *ex vivo* coculture with allogeneic T cells, with preceding culture performed without interferon gamma (-IFNg) or with interferon gamma (+IFNg), for *NPM1* class I samples (left panel; n=7 samples) and *NPM1* class II samples (right panel; n=6 samples). Treatment with IFNg resulted in increased T cell sensitivity for the majority of *NPM1* class I samples as well as *NPM1* class II samples **(c)** Survival of AML cells after three-day *ex vivo* coculture with allogeneic T cells, for *NPM1* class I (n=7samples) and *NPM1* class II samples (n=6 samples), with preceding culture performed without interferon gamma (left panel) or with interferon gamma (+IFNg; right panel). T cell sensitivity was significantly higher in *NPM1* class I samples compared with *NPM1* class II samples both with and without IFNg treatment (two-sided Mann-Whitney U test). Source data are provided as a Source Data file.

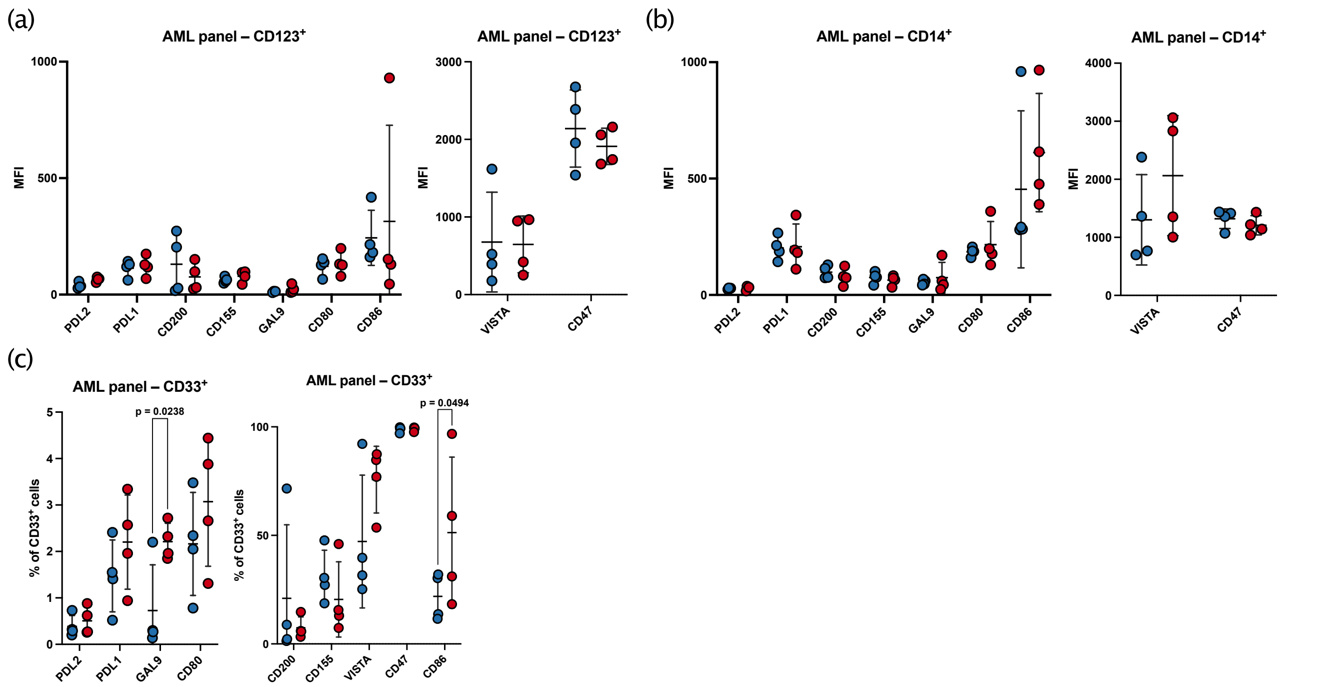

Supplementary Fig. 25 | Checkpoint receptor surface expression on *NPM1*$^{\text{class I}}$ and *NPM1*$^{\text{class II}}$ AML cells. (a) Surface expression of selected checkpoint receptors on immature (CD123+) AML cells from *NPM1*$^{\text{class I}}$ (blue, n=4 samples) and *NPM1*$^{\text{class II}}$ (red, n=4 samples) measured by flow cytometry. **(b)** Surface expression of selected checkpoint receptors on mature (CD14+) AML cells from *NPM1*$^{\text{class I}}$ (blue, n=4 samples) and *NPM1*$^{\text{class II}}$ (red, n=4 samples) measured by flow cytometry. **(c)** Proportion of myeloid (CD33+) AML cells positive for selected checkpoint receptors as measured by flow cytometry for *NPM1*$^{\text{class I}}$ (blue, n=4 samples) and *NPM1*$^{\text{class II}}$ (red, n=4 samples). A significantly higher proportion of *NPM1*$^{\text{class II}}$ myeloid cells are positive for GAL9 expression and CD86 expression compared with *NPM1*$^{\text{class I}}$ cells (two-sided Mann-Whitney U test). Source data are provided as a Source Data file.

(a)

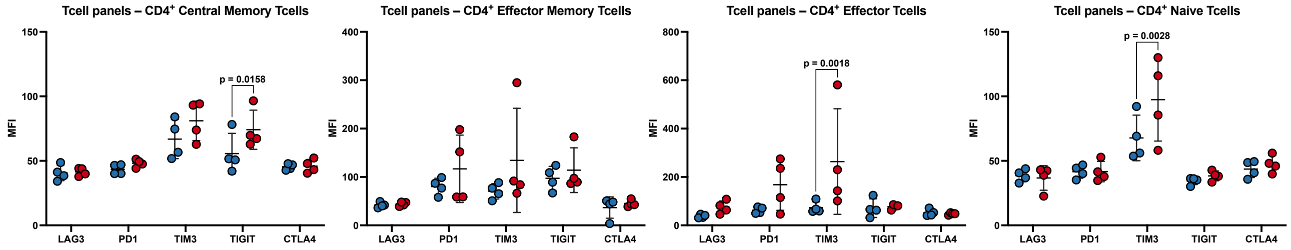

(b)

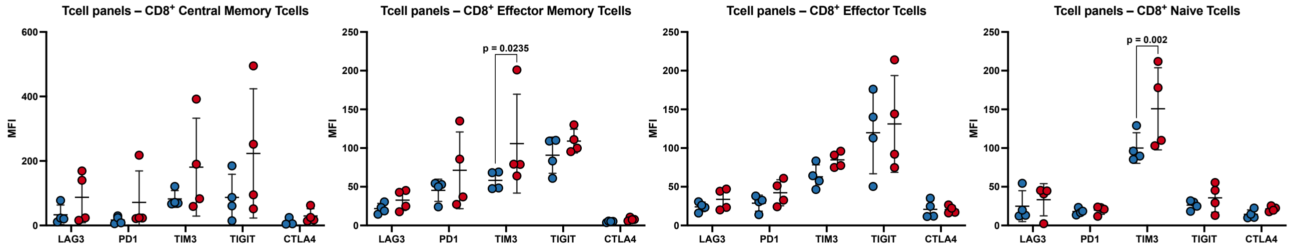

Supplementary Fig. 26 | Checkpoint receptor surface expression on T cell subsets from *NPM1*$^{\text{class I}}$ and *NPM1*$^{\text{class II}}$ diagnostic bone marrow. (a) Surface expression of selected checkpoint molecules on CD4$^+$ central memory T cells (CCR7$^+$CD45RA$^-$; first panel), CD4$^+$ effector memory T cells (CCR7$^-$CD45RA$^-$; second panel), CD4$^+$ T effector memory cells re-expressing CD45RA (TEMRA cells; CCR7$^-$CD45RA$^+$; third panel), and CD4$^+$ naive T cells (CCR7$^+$CD45RA$^+$; fourth panel) from *NPM1*$^{\text{class I}}$ (blue, n=4 samples) and *NPM1*$^{\text{class II}}$ (red, n=4 samples) diagnostic bone marrow, measured by flow cytometry (p-values from two-sided Mann-Whitney U tests). **(b)** Surface expression of selected checkpoint molecules on CD8$^+$ central memory T cells (CCR7$^+$CD45RA$^-$; first panel), CD8$^+$ effector memory T cells (CCR7$^-$CD45RA$^-$; second panel), CD8$^+$ T effector memory cells re-expressing CD45RA (TEMRA cells; CCR7$^-$CD45RA$^+$; third panel), and CD8$^+$ naive T cells (CCR7$^+$CD45RA$^+$; fourth panel) from *NPM1*$^{\text{class I}}$ (blue, n=4 samples) and *NPM1*$^{\text{class II}}$ (red, n=4 samples) diagnostic bone marrow, measured by flow cytometry (p-values from two-sided Mann-Whitney U tests). MFI, geometric mean fluorescence. Source data are provided as a Source Data file.

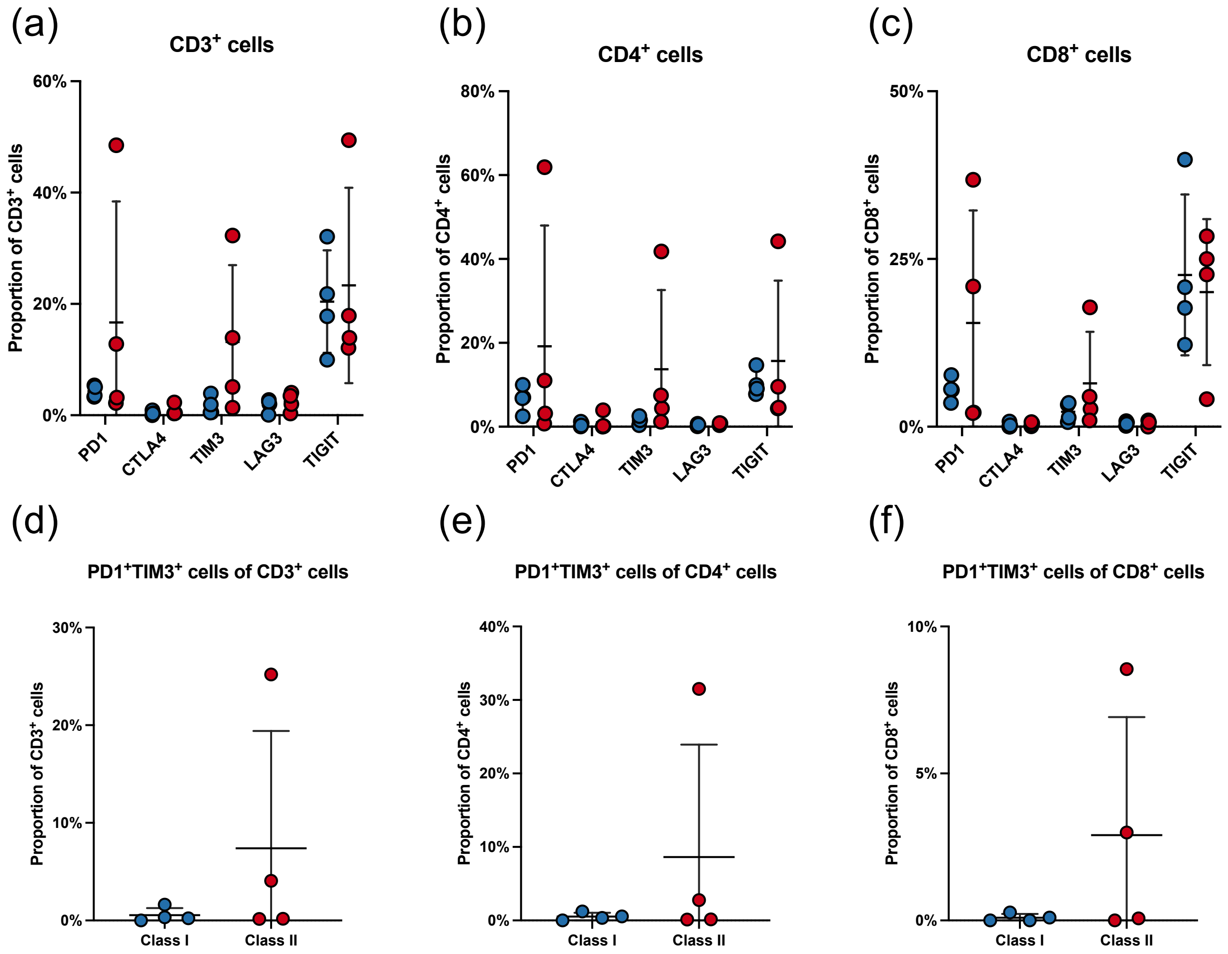

Supplementary Fig. 27 | Proportion of T cells expressing checkpoint receptor combinations in *NPM1*$^{class\ I}$ and *NPM1*$^{class\ II}$ diagnostic bone marrow. (a) Proportion of T cells (CD3+) from AML diagnostic bone marrow positive for selected checkpoint receptors, as measured by flow cytometry for *NPM1*$^{class\ I}$ (blue, n=4 samples) and *NPM1*$^{class\ II}$ (red, n=4 samples). **(b)** Proportion of $CD4^+$ T cells from AML diagnostic bone marrow positive for selected checkpoint receptors, as measured by flow cytometry for *NPM1*$^{class\ I}$ (blue, n=4 samples) and *NPM1*$^{class\ II}$ (red, n=4 samples). **(c)** Proportion of $CD8^+$ T cells from AML diagnostic bone marrow positive for selected checkpoint receptors, as measured by flow cytometry for *NPM1*$^{class\ I}$ (blue, n=4 samples) and *NPM1*$^{class\ II}$ (red, n=4 samples). **(d)** Proportion of T cells (CD3+) from AML diagnostic bone marrow expressing both PD1 and TIM3, indicating an exhausted phenotype, as measured by flow cytometry for *NPM1*$^{class\ I}$ (blue, n=4 samples) and *NPM1*$^{class\ II}$ (red, n=4 samples). **(e)** Proportion of $CD4^+$ T cells from AML diagnostic bone marrow expressing both PD1 and TIM3, indicating an exhausted phenotype, as measured by flow cytometry for *NPM1*$^{class\ I}$ (blue, n=4 samples) and *NPM1*$^{class\ II}$ (red, n=4 samples). **(f)** Proportion of $CD8^+$ T cells from AML diagnostic bone marrow expressing both PD1 and TIM3, indicating an exhausted phenotype, as measured by flow cytometry for *NPM1*$^{class\ I}$ (blue, n=4 samples) and *NPM1*$^{class\ II}$ (red, n=4 samples). Source data are provided as a Source Data file.

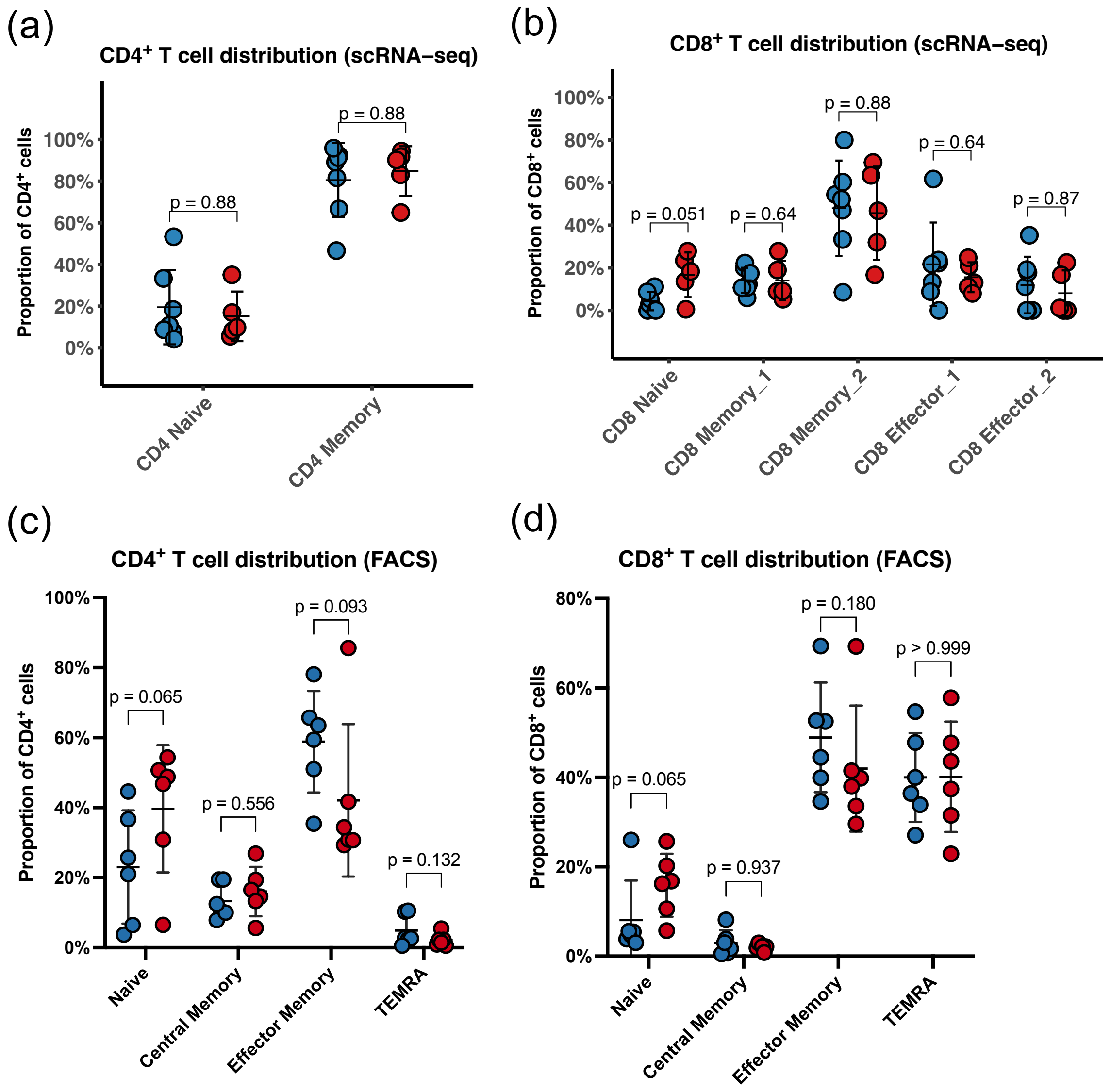

Supplementary Fig. 28 | Proportion of T cell subsets in *NPM1*$^{\text{class I}}$ and *NPM1*$^{\text{class II}}$ samples. (a) Composition of the CD4$^+$ T cell compartment in *NPM1*$^{\text{class I}}$ (blue, n=7 samples) and *NPM1*$^{\text{class II}}$ (red, n=5 samples) BM or PB as determined by scRNA-seq (p-values from two-sided Mann-Whitney U tests). **(b)** Composition of the CD8$^+$ T cell compartment in *NPM1*$^{\text{class I}}$ (blue, n=7 samples) and *NPM1*$^{\text{class II}}$ (red, n=5 samples) BM or PB as determined by scRNA-seq (p-values from two-sided Mann-Whitney U tests). **(c)** Composition of the CD4$^+$ T cell compartment in *NPM1*$^{\text{class I}}$ (blue, n=6 samples) and *NPM1*$^{\text{class II}}$ (red, n=6 samples) BM as determined by flow cytometry (p-values from two-sided Mann-Whitney U tests). **(d)** Composition of the CD8$^+$ T cell compartment in *NPM1*$^{\text{class I}}$ (blue, n=6 samples) and *NPM1*$^{\text{class II}}$ (red, n=6 samples) BM as determined by flow cytometry (p-values from two-sided Mann-Whitney U tests). Source data are provided as a Source Data file.

Supplementary References

1. Cancer Genome Atlas Research Network. Genomic and epigenomic landscapes of adult de novo acute myeloid leukemia. *N. Engl. J. Med.* **368**, 2059–2074 (2013).
2. Tyner, J. W. *et al.* Functional genomic landscape of acute myeloid leukaemia. *Nature* **562**, 526–531 (2018).
3. Papaemmanuil, E. *et al.* Genomic classification and prognosis in acute myeloid leukemia. *N. Engl. J. Med.* **374**, 2209–2221 (2016).
4. Franzén, O., Gan, L.-M. & Björkegren, J. L. M. PanglaoDB: a web server for exploration of mouse and human single-cell RNA sequencing data. *Database* **2019**, baz046 (2019).
5. Bottomly, D. *et al.* Integrative analysis of drug response and clinical outcome in acute myeloid leukemia. *Cancer Cell* **40**, 850-864.e9 (2022).
6. Wang, M. *et al.* Validation of risk stratification models in acute myeloid leukemia using sequencing-based molecular profiling. *Leukemia* **31**, 2029–2036 (2017).
7. Mer, A. S. *et al.* Biological and therapeutic implications of a unique subtype of NPM1 mutated AML. *Nat. Commun.* **12**, 1054 (2021).
