# Supplementary material for: The AML cellular state space unveils NPM1 immune evasion subtypes with distinct clinical outcomes
Source: Nat Commun. 2025 Nov 25;16:10592. doi: 10.1038/s41467-025-66546-6 (PMC12658069; doi:10.1038/s41467-025-66546-6)
Supplement: Supplementary file 2 — Description of Additional Supplementary Files [file 41467_2025_66546_MOESM2_ESM.pdf]

Description of Additional Supplementary Information

Supplementary Data 1. Included AML cases and molecular methods applied.

Supplementary Data 2. Design criteria and primer sequences for scRNAmut-seq reactions.

Supplementary Data 3. Genes used for NPM1 class I/NPM1 class II classification from bulk gene expression.

Supplementary Data 4. Clinical and molecular features of AML cases with NPM1 mutations.

Supplementary Data 5. Clinical features of external AML cases with NPM1 mutations.

Supplementary Data 6. Reduced list of genes for NPM1 class I/NPM1 class II classification.
