# Supplementary material for: The AML cellular state space unveils NPM1 immune evasion subtypes with distinct clinical outcomes
Source: Nat Commun. 2025 Nov 25;16:10592. doi: 10.1038/s41467-025-66546-6 (PMC12658069; doi:10.1038/s41467-025-66546-6)
Supplement: Supplementary file 4 — Reporting Summary [file 41467_2025_66546_MOESM4_ESM.pdf]

nature portfolio

Corresponding author(s): Henrik Lilljebjörn
Thoas Fioretos

Last updated by author(s): Nov 6, 2025

Reporting Summary

Nature Portfolio wishes to improve the reproducibility of the work that we publish. This form provides structure for consistency and transparency in reporting. For further information on Nature Portfolio policies, see our Editorial Policies and the Editorial Policy Checklist.

Statistics

For all statistical analyses, confirm that the following items are present in the figure legend, table legend, main text, or Methods section.

n/a	Confirmed	
☐	☒	The exact sample size (*n*) for each experimental group/condition, given as a discrete number and unit of measurement
☐	☒	A statement on whether measurements were taken from distinct samples or whether the same sample was measured repeatedly
☐	☒	The statistical test(s) used AND whether they are one- or two-sided<br>*Only common tests should be described solely by name; describe more complex techniques in the Methods section.*
☐	☒	A description of all covariates tested
☐	☒	A description of any assumptions or corrections, such as tests of normality and adjustment for multiple comparisons
☐	☒	A full description of the statistical parameters including central tendency (e.g. means) or other basic estimates (e.g. regression coefficient) AND variation (e.g. standard deviation) or associated estimates of uncertainty (e.g. confidence intervals)
☐	☒	For null hypothesis testing, the test statistic (e.g. *F*, *t*, *r*) with confidence intervals, effect sizes, degrees of freedom and *P* value noted<br>*Give P values as exact values whenever suitable.*
☒	☐	For Bayesian analysis, information on the choice of priors and Markov chain Monte Carlo settings
☒	☐	For hierarchical and complex designs, identification of the appropriate level for tests and full reporting of outcomes
☒	☐	Estimates of effect sizes (e.g. Cohen's *d*, Pearson's *r*), indicating how they were calculated

Our web collection on statistics for biologists contains articles on many of the points above.

Software and code

Policy information about availability of computer code

Data collection	No code was used to collect data
Data analysis	The following software was used for analysis of genomic sequencing data:<br>1. BWA (v0.7.15) for alignment.<br>2. Strelka (v0.4.7), Strelka (v2.9.4), Mutect2 (gatk:v4.0.8.1), and freebayes (v1.1.0) for detection of SNVs and small InDels.<br>3. Pindel (v0.2.5b8), manta (v1.4.0), and delly (v0.7.7) for detection of structural variants, larger indels, and tandem repeats.<br>4. cnvkit (v0.9.2) was used for copy number detection.<br>5. snpeff (v0.4.3r) was used for variant annotation.<br>6. R (v4.1.2) was used for visualization.<br><br>RNA-seq analysis:<br>1. RSEM (v1.2.30) was used for gene expression determination.<br>2. chimerascan (v.0.4.5) was used for fusion gene detection.<br>3. Qlucore Omics Explorer (v3.7) was used for gene expression analysis, including hierarchical clustering.<br><br>scRNA-seq analysis:<br>1. Cellranger (v3.1.0) was used for alignment and single cell gene expression determination.<br>2. Seurat (v4.0.0) and R (v4.0.0) was used for data analysis and visualization.<br>3. SingleCellProjections.jl (v0.2.5; https://github.com/BioJulia/SingleCellProjections.jl) was used for data visualization.

4. Qlucore Omics Explorer (v3.7) was used for gene expression analysis of averaged gene expression data over cell types.
5. STAR (v2.7.8a) was used for alignment of single cell mutational data.
6. All custom code to reproduce the analyses supporting this paper is available at https://github.com/rasmushenningsson/AMLStateSpace.

For manuscripts utilizing custom algorithms or software that are central to the research but not yet described in published literature, software must be made available to editors and reviewers. We strongly encourage code deposition in a community repository (e.g. GitHub). See the Nature Portfolio guidelines for submitting code & software for further information.

Data

Policy information about availability of data

All manuscripts must include a data availability statement. This statement should provide the following information, where applicable:

- Accession codes, unique identifiers, or web links for publicly available datasets
- A description of any restrictions on data availability
- For clinical datasets or third party data, please ensure that the statement adheres to our policy

The publicly available data used in this study are available from Genomic Data Commons as study laml_2012 [https://gdc.cancer.gov/about-data/publications/laml_2012] (TCGA9 SNV and indel data), from the cBioPortal for Cancer Genomics (https://www.cbioportal.org/datasets) as studies TCGA, NEJM 2013 [https://cbioportal-datahub.s3.amazonaws.com/laml_tcga_pub.tar.gz] and OHSU, Nature 2018 [https://cbioportal-datahub.s3.amazonaws.com/aml_ohsu_2018.tar.gz] (TCGA9 and Beat-AML131 gene expression data), from Vizome as study BeatAML [http://www.vizome.org/aml/geneset/] (Beat-AML131 SNV and indel data), from Github repositories BeatAML2 [https://biodev.github.io/BeatAML2/] (Beat-AML234 gene expression data) and AML-multistage [https://github.com/gerstung-lab/AML-multistage/tree/master/data] (Papaemmanuil et al10, SNV and indel data), and from Zenodo as study Clinseq_AML [https://zenodo.org/records/292986] (Clinseq35 gene expression data). The raw sequencing data generated in this study have been deposited in the European Genome-Phenome Archive (EGA; https://ega-archive.org/) under the accession codes: EGAD50000001574 (https://ega-archive.org/datasets/EGAD50000001574; MP-WGS data), EGAD50000001575 (https://ega-archive.org/datasets/EGAD50000001575; WES data), EGAD50000001576 (https://ega-archive.org/datasets/EGAD50000001576; RNA-seq data), and EGAD50000001577 (https://ega-archive.org/datasets/EGAD50000001577; scRNA-seq data). The raw sequencing data deposited at EGA are available under restricted access due to the General Data Protection Regulation (GDPR), the Swedish data protection legislation and the Swedish Public Access to Information and Secrecy Act. Access can be obtained by contacting request@researchdata.lu.se. Access will be granted for projects ensuring data protection in compliance with the aforementioned legislation which can be further specified in a data access agreement provided upon request. The first response after requests for access are expected to occur within five business days. Once access has been granted, data will be available for the duration of the specified project. The processed gene expression data are available as gene expression matrices from the ScilifeLab Data Repository (https://figshare.scilifelab.se/) through the following DOIs: https://doi.org/10.17044/scilifelab.2155716372 (RNA-seq data), https://doi.org/10.17044/scilifelab.2371564873 (scRNA-seq data). The remaining data are available within the Article, Supplementary Information or Source Data file. Source Data are provided with this paper.

Research involving human participants, their data, or biological material

Policy information about studies with human participants or human data. See also policy information about sex, gender (identity/presentation), and sexual orientation and race, ethnicity and racism.

Reporting on sex and gender	Primary leukemia samples (n=112) were collected from both male (n=63) and female (n=49) patients (based on Swedish personal identification numbers) to allow for equitable implementation of clinically relevant findings.
Reporting on race, ethnicity, or other socially relevant groupings	The study does not contain any data on socially constructed or socially relevant categorization variables.
Population characteristics	The study population consists of adult AML patients from a consecutive cohort of cases, between 18-86 years of age. Inclusion relied solely on the basis of genetic aberrations and sample availability in order to obtain a cohort with adequate cell numbers and sufficient sample sizes for all major genetic subtypes. No selection was performed based on disease history, treatment, age, sex/gender or other variables. The patient characteristics are detailed in Supplementary Data 1.
Recruitment	Samples were selected from a cohort of consecutive AML patients diagnosed at the Skåne University Hospital in Lund, based solely sample availability. Patient inclusion was dependent on informed consent, which could bias the cohort towards patients amenable to research perticipation. However, this is unlikely to correlate with the somatic genetic aberrations studied in this project and impact the results of the study. Healthy bone marrow donors received modest financial compensation. No other compensation was awarded for study participation.
Ethics oversight	The research complies with all relevant ethical regulations. Primary samples were collected at Skåne university hospital after written informed consent from patients and in accordance with the Declaration of Helsinki. Experiments with primary leukemia samples were approved by the Swedish Ethical Review Authority (dnr 2011/289 and dnr 2023-01550-01).

Note that full information on the approval of the study protocol must also be provided in the manuscript.

Field-specific reporting

Please select the one below that is the best fit for your research. If you are not sure, read the appropriate sections before making your selection.

[x] Life sciences [ ] Behavioural & social sciences [ ] Ecological, evolutionary & environmental sciences

For a reference copy of the document with all sections, see nature.com/documents/nr-reporting-summary-flat.pdf

Life sciences study design

All studies must disclose on these points even when the disclosure is negative.

Sample size	No statistical analysis was used to predetermine the sample size. Sample size was determined by the availability of material, cost of experiments, and the length of the inclusion period.
Data exclusions	No data was excluded from the study.
Replication	To ensure reproducability, experiments were repeated as described in the manuscript. The finding of two distinct gene expression profiiles within the immature cells of the NPM1 subtype was sucessfully reproduced in three external RNA-seq datasets, although a third of the external samples did not contain either of the two expression profiles, possibly due to low contents of immature cells in these samples.
Randomization	All available samples were used in the initial cohort (n=120). For scRNA-seq analysis, samples (n=38) were selected to be representative of the overall cohort with regard to sample subtype.
Blinding	Group allocations were performed after WES, MP-WGS and RNA-seq analysis, and were therefore unknown when this data were analyzed. After molecular subtypes were identified, sample IDs were known to the investigators to keep track of samples used in the experiments.

Reporting for specific materials, systems and methods

We require information from authors about some types of materials, experimental systems and methods used in many studies. Here, indicate whether each material, system or method listed is relevant to your study. If you are not sure if a list item applies to your research, read the appropriate section before selecting a response.

Materials & experimental systems

n/a	Involved in the study	
☐	☒	Antibodies
☒	☐	Eukaryotic cell lines
☒	☐	Palaeontology and archaeology
☒	☐	Animals and other organisms
☒	☐	Clinical data
☒	☐	Dual use research of concern
☒	☐	Plants

Methods

n/a	Involved in the study	
☒	☐	ChIP-seq
☐	☒	Flow cytometry
☒	☐	MRI-based neuroimaging

Antibodies

Antibodies used

PE/Cy7 anti-C3AR (BioLegend, clone: hC3aRZ8, cat# 345808, lot# B313054, dilution: 5µl in 100µl)
PE/Cy7 anti-CCR7 (BioLegend, clone: G04347, cat# 353226, lot# B399690, dilution: 5µl in 100µl)
AF488 anti-CD117 (BioLegend, clone: 104D2, cat# 313234, lot# B225861, dilution: 1µl in 100µl)
BV711 anti-CD123 (BD Biosciences, clone: 9F5, cat# 563161, lot# 4038703, dilution: 1µl in 100µl)
BV711 anti-CD123 (BioLegend, clone: 6H6, cat# 306030, lot# B391531, dilution: 1µl in 100µl)
APC anti-CD14 (BioLegend, clone: M5E2, cat# 301808, lot# B371106, dilution: 1µl in 100µl)
BV421 anti-CD155 (BioLegend, clone: SKII.4, cat# 337631, lot# B391600, dilution: 5µl in 100µl)
APC/Cy7 anti-CD19 (BioLegend, clone: HIB19, cat# 302218, lot# B279663, dilution: 3µl in 100µl)
BV510 anti-CD19 (BioLegend, clone: HIB19, cat# 302242, lot# B399472, dilution: 1.5µl in 100µl)
PE/Cy7 anti-CD200 (BioLegend, clone: A1804213, cat# 399805, lot# B390652, dilution: 5µl in 100µl)
PE anti-CD25 (BioLegend, clone: BV96, cat# 302606, lot# B354945, dilution: 5µl in 100µl)
PE/Cy7 anti-CD3 (BioLegend, clone: SK7, cat# 344816, lot# B285813, dilution: 1µl in 100µl)
BV510 anti-CD3 (BioLegend, clone: OKT3, cat# 317331, lot# B384771, dilution: 1.5µl in 100µl)
PE anti-CD3 (BioLegend, clone: OKT3, cat# 317308, lot# B387153, dilution: 1.5µl in 100µl)
BV421 anti-CD33 (BioLegend, clone: WM53, cat# 303416, lot# B337161, dilution: 1µl in 100µl)
PE anti-CD33 (BioLegend, clone: WM53, cat# 303404, lot# B340162, dilution: 1µl in 100µl)
APC/Cy7 anti-CD33 (BioLegend, clone: WM53, cat# 303442, lot# B376706, dilution: 1.5µl in 100µl)
AF488 anti-CD34 (BioLegend, clone: 581, cat# 343518, lot# B280731, dilution: 3µl in 100µl)
APC/Cy7 anti-CD38 (BioLegend, clone: HIT2, cat# 303534, lot# B345003, dilution: 3µl in 100µl)
BV711 anti-CD4 (BioLegend, clone: SK3, cat# 344648, lot# B399745, dilution: 1µl in 100µl)
BV510 anti-CD45RA (BioLegend, clone: HI100, cat# 304142, lot# B410165, dilution: 3µl in 100µl)
BV421 anti-CD47 (BioLegend, clone: CC2C6, cat# 323115, lot# B415202, dilution: 5µl in 100µl)
APC anti-CD69 (BioLegend, clone: FN50, cat# 310910, lot# B359249, dilution: 5µl in 100µl)
APC/Cy7 anti-CD8 (BioLegend, clone: SK1, cat# 344714, lot# B391918, dilution: 1µl in 100µl)
FITC anti-CD80 (BioLegend, clone: 2D10, cat# 305205, lot# B362201, dilution: 5µl in 100µl)
PE anti-CD86 (BioLegend, clone: BU63, cat# 374205, lot# B357248, dilution: 5µl in 100µl)

BV421 anti-CTLA4 (BioLegend, clone: BNI3, cat# 369605, lot# B400665, dilution: 3µl in 100µl)
PE/Cy7 anti-GAL9 (BioLegend, clone: 9M1-3, cat# 348915, lot# B397671, dilution: 5µl in 100µl)
APC anti-GPR56 (BioLegend, clone: 4C3, cat# 391906, lot# B295891, dilution: 1µl in 100µl)
BV421 anti-HLA-DR,DP,DQ (BD Biosciences, clone: Tu39, cat# 564244, lot# 1154491, dilution: 1µl in 100µl)
APC anti-LAG3 (BioLegend, clone: 7H2C65, cat# 369211, lot# B401587, dilution: 3µl in 100µl)
BV421 anti-Mouse IgG1k (BioLegend, clone: MOPC-173, cat# 400259, lot# B206805, dilution: 1µl in 100µl)
BV421 anti-PD1 (BioLegend, clone: EH12.2H7, cat# 329919, lot# B373432, dilution: 3µl in 100µl)
FITC anti-PDL1 (BioLegend, clone: MIH2, cat# 393605, lot# B418572, dilution: 5µl in 100µl)
BV421 anti-PDL2 (BioLegend, clone: MIH18, cat# 345519, lot# B392346, dilution: 5µl in 100µl)
APC anti-TIGIT (BioLegend, clone: A151539, cat# 372705, lot# B393085, dilution: 3µl in 100µl)
FITC anti-TIM3 (BioLegend, clone: F38-2E2, cat# 345021, lot# B393335, dilution: 3µl in 100µl)
PE anti-VISTA (BD Biosciences, clone: MIH65.rMAb, cat# 56672, lot# 3212057, dilution: 5µl in 100µl)
TruStain FcX, Human (BioLegend, clone: –, cat# 422302, lot# B340501, dilution: 3µl in 100µl)

Validation

All antibodies were commercially available from either BioLegend or BD Biosciences and were validated by the manufacturer, see https://www.biolegend.com/en-gb/quality/quality-control and https://www.bdbiosciences.com/en-us/products/reagents/flow-cytometry-reagents/research-reagents/quality-and-reproducibility

Plants

Seed stocks

Report on the source of all seed stocks or other plant material used. If applicable, state the seed stock centre and catalogue number. If plant specimens were collected from the field, describe the collection location, date and sampling procedures.

Novel plant genotypes

Describe the methods by which all novel plant genotypes were produced. This includes those generated by transgenic approaches, gene editing, chemical/radiation-based mutagenesis and hybridization. For transgenic lines, describe the transformation method, the number of independent lines analyzed and the generation upon which experiments were performed. For gene-edited lines, describe the editor used, the endogenous sequence targeted for editing, the targeting guide RNA sequence (if applicable) and how the editor was applied.

Authentication

Describe any authentication procedures for each seed stock used or novel genotype generated. Describe any experiments used to assess the effect of a mutation and, where applicable, how potential secondary effects (e.g. second site T-DNA insertions, mosiacism, off-target gene editing) were examined.

Flow Cytometry

Plots

Confirm that:

☒ The axis labels state the marker and fluorochrome used (e.g. CD4-FITC).

☒ The axis scales are clearly visible. Include numbers along axes only for bottom left plot of group (a 'group' is an analysis of identical markers).

☒ All plots are contour plots with outliers or pseudocolor plots.

☒ A numerical value for number of cells or percentage (with statistics) is provided.

Methodology

Sample preparation

Bone marrow aspirates and peripheral blood samples were collected at Skåne university hospital after written informed consent in accordance to the Declaration of Helsinki. Samples were collected from patients with AML and healthy controls. Mononuclear cells were isolated using Lymphoprep (GE Healthcare, Sweden) and viably frozen before thawing and staining for flow cytometry. Bone marrow or peripheral blood mononuclear cells from NPM1 class I AML cases (n=11), NPM1 class II AML cases (n=8) and healthy normal bone marrow (NBM) donors (n=4) were stained at 4 degrees C for 20 minutes and subsequently washed and resuspended for flow cytometry on an LSR Fortessa (BD Biosciences, USA).

Instrument

BD LSR fortessa

Software

BD FACSDiva was used for cell acquisition and FlowJo 10 was used for post-acquisition analysis.

Cell population abundance

0.2-1 million cells were used for flow cytometry. The AML immature population comprised between 0.3-51 % of the samples and was determined based on markers defined by scADT-seq. When this was not available, the AML immature population was assumed to be CD33+CD117+

Gating strategy

1) 7-AAD vs FSC-A. 2) SSC-A vs FSC-A. 3) FSC-H vs FSC-A. Then, combinations of CD33, CD117, CD123, and/or C3AR, as defined by scADT-seq, were used to define the AML immature population. The gating strategy of each individual sample is presented in Supplementary Figs 21-22.

☒ Tick this box to confirm that a figure exemplifying the gating strategy is provided in the Supplementary Information.
